# Antitumoral Activity of the Universal Methyl Donor *S*-Adenosylmethionine in Glioblastoma Cells

**DOI:** 10.3390/molecules29081708

**Published:** 2024-04-10

**Authors:** Laura Mosca, Cristina Pagano, Roberta Veglia Tranchese, Roberta Grillo, Francesca Cadoni, Giovanna Navarra, Laura Coppola, Martina Pagano, Luigi Mele, Giovanna Cacciapuoti, Chiara Laezza, Marina Porcelli

**Affiliations:** 1 Department of Precision Medicine, University of Campania “Luigi Vanvitelli”, Via Luigi De Crecchio 7, 80138 Naples, Italy; laura.mosca@unicampania.it (L.M.); roberta.vegliatranchese@unicampania.it (R.V.T.); roberta.grillo@unicampania.it (R.G.); francesca.cadoni@unicampania.it (F.C.); martina.pagano@unicampania.it (M.P.); marina.porcelli@unicampania.it (M.P.); 2 Department of Molecular Medicine and Medical Biotechnology, University of Naples “Federico II”, Via Pansini 5, 80131 Naples, Italy; pagano.cris@gmail.com (C.P.); vanna.navarra@libero.it (G.N.); coppola.laura6@gmail.com (L.C.); 3 Department of Experimental Medicine, University of Campania “Luigi Vanvitelli”, Via Luciano Armanni 5, 80138 Naples, Italy; luigi.mele@unicampania.it; 4 Institute of Endocrinology and Experimental Oncology (IEOS), National Research Council (CNR), Via Pansini 5, 80131 Naples, Italy; c.laezza@ieos.cnr.it

**Keywords:** *S*-Adenosylmethionine, glioblastoma, cell cycle arrest and apoptosis, DNA damage response, homologous recombination repair, mitotic catastrophe

## Abstract

Glioblastoma (GBM), the most frequent and lethal brain cancer in adults, is characterized by short survival times and high mortality rates. Due to the resistance of GBM cells to conventional therapeutic treatments, scientific interest is focusing on the search for alternative and efficient adjuvant treatments. *S*-Adenosylmethionine (AdoMet), the well-studied physiological methyl donor, has emerged as a promising anticancer compound and a modulator of multiple cancer-related signaling pathways. We report here for the first time that AdoMet selectively inhibited the viability and proliferation of U87MG, U343MG, and U251MG GBM cells. In these cell lines, AdoMet induced S and G2/M cell cycle arrest and apoptosis and downregulated the expression and activation of proteins involved in homologous recombination DNA repair, including RAD51, BRCA1, and Chk1. Furthermore, AdoMet was able to maintain DNA in a damaged state, as indicated by the increased γH2AX/H2AX ratio. AdoMet promoted mitotic catastrophe through inhibiting Aurora B kinase expression, phosphorylation, and localization causing GBM cells to undergo mitotic catastrophe-induced death. Finally, AdoMet inhibited DNA repair and induced cell cycle arrest, apoptosis, and mitotic catastrophe in patient-derived GBM cells. In light of these results, AdoMet could be considered a potential adjuvant in GBM therapy.

## 1. Introduction

Glioblastoma (GBM), previously known as glioblastoma multiforme, a grade IV astrocytoma, is the most aggressive and common primary brain tumor in adults and one of the deadliest human cancers, accounting for 4% of all cancer deaths with a 5-year survival rate of 5.1% [1,2]. Despite an aggressive, multimodal treatment including surgery followed by radiation and chemotherapy with the alkylating agent temozolomide [3,4], the prognosis for patients with GBM continues to be limited with a median survival ranging from 14 to 30 months after diagnosis, depending on the molecular subtype of the tumor [5,6]. Therapeutic resistance in GBM is due to the ability of cancer cells to overcome DNA damage caused by radiation and chemotherapeutic agents through the activation of the DNA damage response (DDR) responsible for cancer progression and recurrence [7,8,9]. GBM harbors many genetic alterations interfering with cancer-related pathways, such as cell growth control, apoptosis, angiogenesis, and cell invasion [10]. Epigenetic alterations, including the hypomethylation of broad regions of DNA occurring mainly at the level of gene promoters, are also involved, alone or in combination with genetic mechanisms, in GBM progression, leading to the activation of oncogenes and pro-metastatic genes [11]. What is noteworthy is that the hypomethylation of the *O*-6-methylguanine-DNA methyltransferase (MGMT) gene results in the increased expression of this DNA repair enzyme, causing resistance to temozolomide [12]. Interestingly, the epigenetic silencing of MGMT via promoter methylation has been shown to be related to prolonged survival in GBM patients treated with alkylating agents [13]. Notably, no therapeutic drugs for DNA hypomethylation are currently FDA-approved or in clinical trials. In this scenario, natural compounds such as *S*-adenosylmethionine (AdoMet), the physiological methyl donor able to regulate gene expression through epigenetic mechanisms involving modifications and alterations occurring at transcriptional and/or posttranscriptional levels, could be exploited as therapeutic drugs to improve the sensitivity of GBM cells to the conventional therapies.

**Figure 1:**
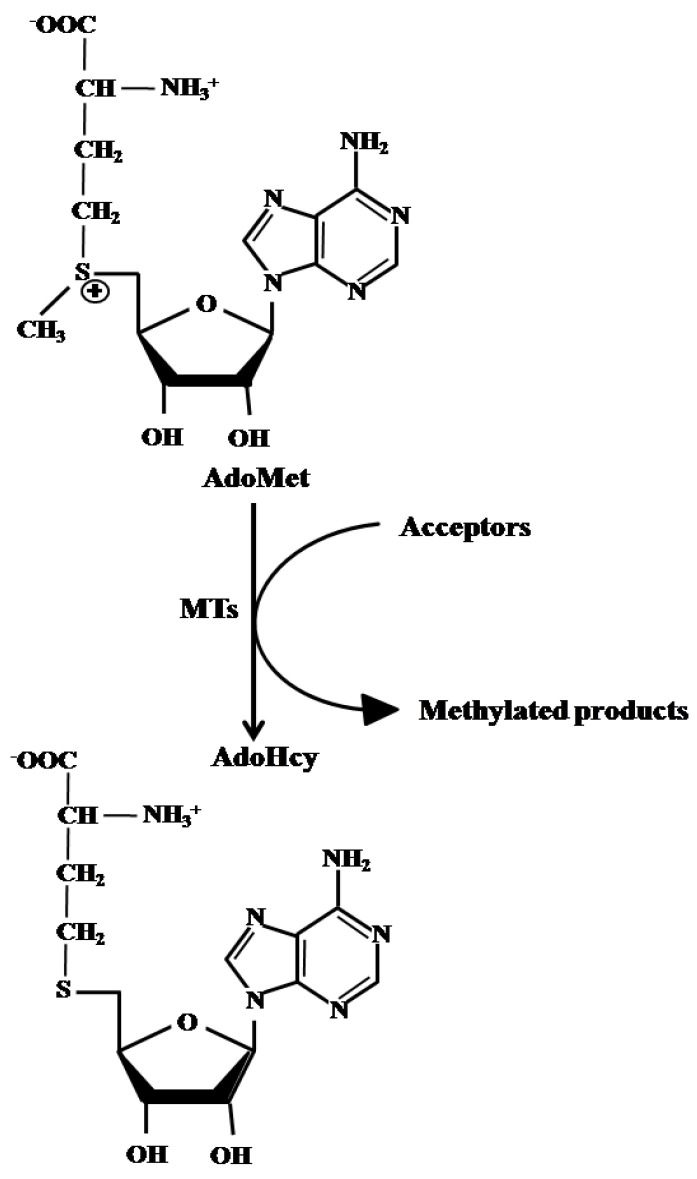
Schematic representation of AdoMet-dependent transmethylation reactions. MTs: methyltransferases.

AdoMet is a naturally occurring sulfonium compound present in all mammalian cells where it plays a wide variety of well-documented biological functions [14,15,16]. AdoMet is the most extensively used enzyme cofactor after ATP due to the large number of physiological processes in which it is involved, being a key player in transmethylation, transsulfuration, and aminopropylation pathways [14,15,16]. Notably, AdoMet can be freely transferred from the cytoplasm to nucleus through nuclear pore complexes [17] and plays a regulatory role in the nucleus, where it participates in histone methylation [18]. All AdoMet-dependent transmethylation reactions produced, as a by-product, S-adenosylhomocysteine (AdoHcy) (Figure 1), a sulfur-containing nucleoside which acts as a potent competitive inhibitor of methyltransferases, thus representing an important regulatory factor. The relative levels of AdoMet and AdoHcy are normally tightly regulated in cells. The intracellular AdoMet/AdoHcy ratio, also defined as the “methylation index”, is considered a reliable indicator of the flow of methyl groups transferred from AdoMet to methyl acceptors within the cells and represents a potent determinant in the regulation of chromatin methylation [19]. In particular, it has been proposed that the inhibition of MGMT expression by alterations in the AdoMet/AdoHcy ratio could be used as a novel pharmacological strategy to improve the responsiveness of GBM cell lines to alkylating agents [20]. To date, AdoMet is a FDA-approved therapeutic agent known to cause DNA hypermethylation and the silencing of hypomethylated genes in cancer cells.

Although the chemical structure of AdoMet was described, for the first time, in the 1950s by Giulio Cantoni, the promising anticancer properties of this physiological compound have been evidenced only in the past two decades by several in vitro and in vivo studies. The potential of AdoMet as an anticancer agent has stimulated growing scientific interest taking advantage of its pleiotropic effects on different survival pathways known to be important in carcinogenesis. Recently, the antiproliferative properties of AdoMet and its involvement in multiple cellular processes including proliferation, differentiation, cell cycle regulation, and apoptosis in various cancer cells have been widely reported in the literature [16,21,22,23,24,25,26,27,28,29,30].

Epigenetic modifications are gaining strong relevance in GBM because they can be both clinical biomarkers and potential drug targets as suggested by many preclinical studies. In recent decades, epigenetic aberrations in GBM have become an attractive topic for the identification and development of new therapeutic strategies since DNA methylation, histone modifications, chromatin remodeling, and aberrant microRNA expression are widely involved in tumorigenesis and tumor-related mechanisms. Most of the researches regarding epigenetic alterations in GBM have drawn particular attention to the role of DNA methylation, such as genome-wide hypomethylation, gene-specific hypomethylation, and hypermethylation [31]. The contribution of epigenetic changes to glioblastoma pathology has been broadly studied in terms of the aberrant promoter methylation-induced silencing of several genes involved in key cellular functions such as the cell cycle, DNA repair, tumor invasion, and apoptosis [32]. Interestingly, it has been recently reported that in two different breast cancer cell lines, AdoMet treatment differentially regulated genes implicated in cancer progression and metastasis and changed the promoter methylation status and protein expression of prometastatic genes [24]. Moreover, as highlighted by the analysis of methylome promoters and enhancers, AdoMet is able to hypermethylate and downregulate genes in pathways of growth and metastasis that are upregulated in liver cancer cells [33]. Furthermore, in MGC-803 human gastric cancer cells and in HT-29 colon carcinoma cells, the treatment with AdoMet leads to methylation in the promoter of c-myc and H-ras oncogenes inhibiting protein expression and resulting in reduced tumorigenesis. Interestingly, treatment with AdoMet did not induce changes in the expression of c-myc and H-ras in non-cancerous cells [34].

Many experimental findings have highlighted the therapeutical potential of AdoMet as an effective adjuvant in conventional cancer chemotherapy to overcome drug resistance [26,27,28,29]. Increasing evidence has also demonstrated that the epigenetic regulation of oncogenic or tumor-suppressor microRNAs represents one of the main mechanisms underlying the anticancer activity of AdoMet [30,35,36,37,38].

In the current study, we reported the anticancer effects of AdoMet on human GBM U87MG, U343MG, and U251MG cells lines and on patient-derived human GBM cells, and we explored the underlying mechanisms. We provided evidence that AdoMet inhibited GBM cell viability and proliferation by inducing S and G2/M cell cycle arrest and apoptosis and by targeting crucial growth and survival pathways. We also demonstrated that AdoMet targeted DNA repair, cell cycle checkpoint activation, and spindle assembly processes leading to mitotic catastrophe-induced cell death. Taken together, these findings suggest that AdoMet may be a promising adjuvant to potentiate current therapies against GBM.

## 2. Results

### 2.1. AdoMet Inhibited GBM Cell Viability

**Figure 2:**
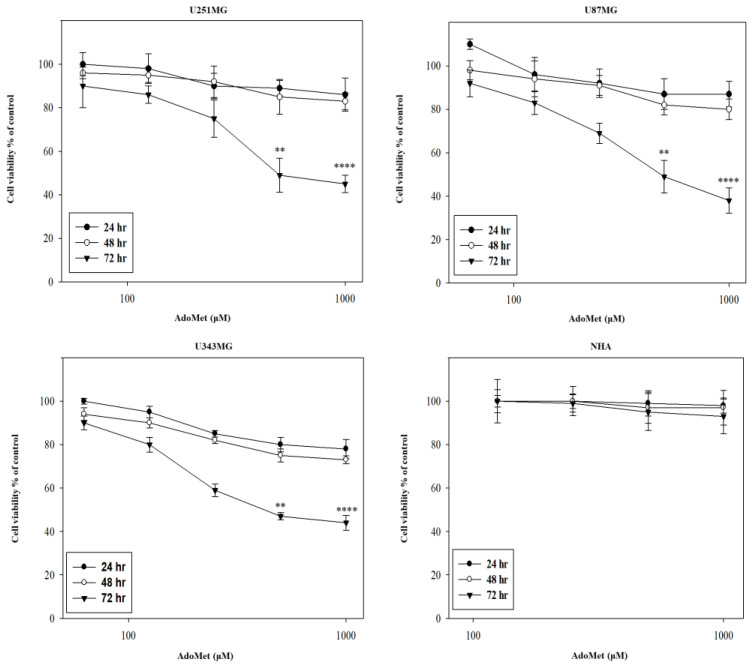
Effect of AdoMet on cell viability of GBM cells. U251MG, U87MG, U343MG, and NHA cell lines were treated or not (control) with increasing amounts of AdoMet (72–1000 μM) (X axis in log scale) for 24, 48, and 72 h. Cell viability was then measured by MTT assay and expressed as a percentage of the control cells. Error bars depict the standard deviation (SD) of triplicated measurements and are representative of three separate experiments, ** *p* < 0.01, **** *p* < 0.001 versus untreated cells.

To investigate the antitumor activity of AdoMet in GBM, U87MG, U251MG, and U343MG cell lines were incubated for 24, 48, and 72 h with increasing concentrations of AdoMet ranging from 72 μM to 1000 μM, and cell proliferation was then assessed by an MTT assay. The results obtained evidenced that AdoMet treatment significantly reduced cell viability in a dose- and time-dependent manner (Figure 2). It has to be noted that after 24 and 48 h, AdoMet at 1000 μM concentration exerted only a weak cytotoxic effect while a prolonged incubation time resulted in a large loss of cell viability with an IC50 value of about 500 μM at 72 h. Parallel direct cell counting provided similar results. Based on these findings, we selected, for further investigations, an incubation time of 72 h. Comparative experiments carried out with NHA showed no significant inhibitory effect on cell viability even at the highest AdoMet concentration. This evidence clearly indicates that AdoMet is able to effectively damage GBM tumor cells, thus confirming the high safety profile of this physiological compound in normal cells, as described in the literature [24]. For this reason, we decided not to use human astrocytes for further investigation.

### 2.2. AdoMet Induced S and G2/M Cell Cycle Arrest

**Figure 3:**
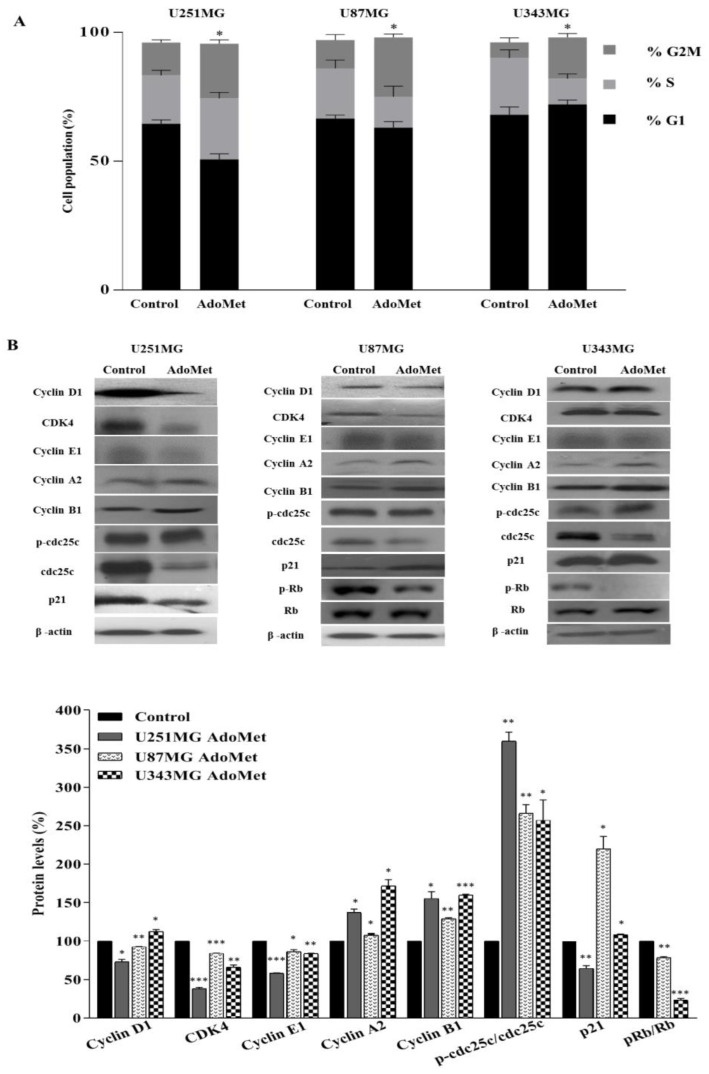
Effects of AdoMet on cell cycle distribution in GBM cells. (**A**) U251MG, U87MG, and U343MG cells were cultured for 72 h in medium supplemented or not (control) with AdoMet 500 μM, and the cell cycle was then assessed by flow cytometry. Histograms show the percentage of cells in each phase of cell cycle. At least 2 × 10^4^ events were analyzed for each sample. Data represent the average of three independent experiments. Error bars depict the SDs. * *p* < 0.05 versus untreated cells. (**B**) The levels of the main cell cycle regulatory proteins were evaluated by Western blot, and the relative densitometric analyses performed for each protein in relation to its relative housekeeping protein were reported as a percentage of untreated cells (100%). Uncropped images of Western blots and their housekeeping proteins are reported in Figures S1–S3. Representative housekeeping β-actin protein used as a loading control was shown. Error bars represent the SD. * *p* < 0.05, ** *p* < 0.01, *** *p* < 0.005 versus untreated cells. The images are representative of three immunoblotting analyses obtained from three independent experiments.

To identify the underlying mechanism of AdoMet-mediated growth inhibition, the cell cycle distribution was analyzed by flow cytometry in U87MG, U251MG, and U343MG cells treated for 72 h with 500 μM AdoMet. As shown in Figure 3A, AdoMet induced a remarkable arrest at G2/M in the three GBM cell lines, evidenced by the significant increase in cell percentage in this phase of the cell cycle with respect to untreated cells (from 12.7% to 21.2% in U251MG, from 11.0% to 23.0% in U87MG, and from 6.1% to 16.0% in U343MG cells). A concomitant decrease in the S population was observed in U87MG (from 19.5% to 12.0%) and in U343MG (from 22% to 10%) cells. On the contrary, the accumulation of cells in the S phase compared to the control (from 18.8% to 23.7%) observed in U251MG cells indicated that AdoMet was able to delay U251MG cell cycle progression in both S and G2/M phases. To gain further insight into the molecular mechanism of AdoMet-induced cell cycle arrest, we analyzed the expression level of some relevant cell proliferation and cell cycle regulators by Western blot. As shown in Figure 3B, the increase in cyclin B1 and cyclin A2 and the decrease in cyclin E1 were observable in AdoMet-treated GBM cells, while cyclin D1 appeared downregulated in U251MG and U87MG cells and slightly upregulated in U343MG cells. Notably, D-type cyclins and their associated cyclin-dependent kinase CDK4 are key components of cell cycle machinery in driving the G1 to S phase transition via phosphorylating and inactivating the retinoblastoma (RB) protein [39]. We therefore examined the protein levels of CDK4 and RB proteins. The results obtained showed a decrease in CDK4, to different extents, in the three GBM cell lines, associated with reduced levels of the phosphorylated form of the RB protein in U87MG and U343MG cells, confirming the inhibitory effect of AdoMet on cell cycle progression. We then analyzed the cell cycle inhibitor p21. Interestingly, we found that after AdoMet treatment, p21 content increased in U343MG and most markedly in U87MG cells, while it became downregulated in U251MG cells, a GBM cell line harboring a TP53 gene mutation [40], suggesting that AdoMet-induced cell cycle arrest in U251MG cells probably involve a p53/p21-independent process.

Finally, our results showed a substantial increase in the levels of phosphorylated cell division cycle 25c (cdc25c) phosphatase (Figure 3B). Cdc25c is a crucial cell cycle regulatory protein that activates the cyclin B1/CDK1 complex in cells for entering mitosis. G2/M checkpoint is largely mediated through the phosphorylation of cdc25c that downregulates the phosphatase activity [41].

### 2.3. AdoMet Induced Apoptosis

**Figure 4:**
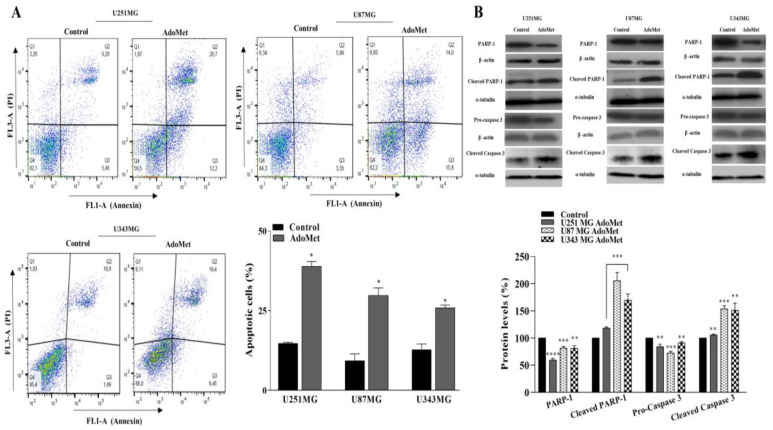
Effects of AdoMet on apoptosis in GBM cells. (**A**) U251MG, U87MG, and U343MG cells were cultured for 72 h in medium supplemented or not (control) with AdoMet 500 μM, and the apoptotic process was then assessed by FACS analysis. Histograms show the percentage of apoptotic cells. For each sample, at least 2 × 10^4^ events were analyzed. Data represent the average of three independent experiments. Error bars depict the SDs. * *p* < 0.05 versus untreated cells. (**B**) The protein levels of uncleaved caspase-3 and PARP-1 and the corresponding cleaved forms were evaluated by Western blot, and the relative densitometric analyses are reported as percentage of untreated control (100%). Housekeeping proteins, used as a loading control, were reported. Error bars represent the SDs. ** *p* < 0.01 ***, *p* < 0.005, **** *p* < 0.001 versus untreated cells. The images are representative of three immunoblotting analyses obtained from three independent experiments. Uncropped images of Western blots and their housekeeping proteins are reported in Figures S4–S6.

The occurrence of apoptosis in U87MG, U251MG, and U343MG cells upon treatment with 500 μM AdoMet was assessed by flow cytometry. As shown in Figure 4A, after 72 h treatment, a significant increase in the percentage of apoptotic cells with values approximately 24%, 20%, and 13% higher than the control was observed in U251MG, U87MG, and U343MG cells, respectively, indicating that AdoMet effectively induced apoptosis in GBM cells. To verify the above findings, the effect of AdoMet on the activation of caspase 3 and PARP-1, a known target of apoptosis-associated caspase cleavage, was analyzed by Western blot. As shown in Figure 3B, AdoMet treatment caused the decrease in pro-caspase 3 and the PARP-1 protein while the levels of cleaved caspase 3 and cleaved PARP-1 significantly increased in treated cells compared to the control, confirming the AdoMet-induced activation of the apoptotic process. All the data above furnished evidence that AdoMet could efficiently cause cell death in GBM cells by inducing apoptosis.

### 2.4. AdoMet Downregulated the Proteins Involved in Homologous Recombination Repair

**Figure 5:**
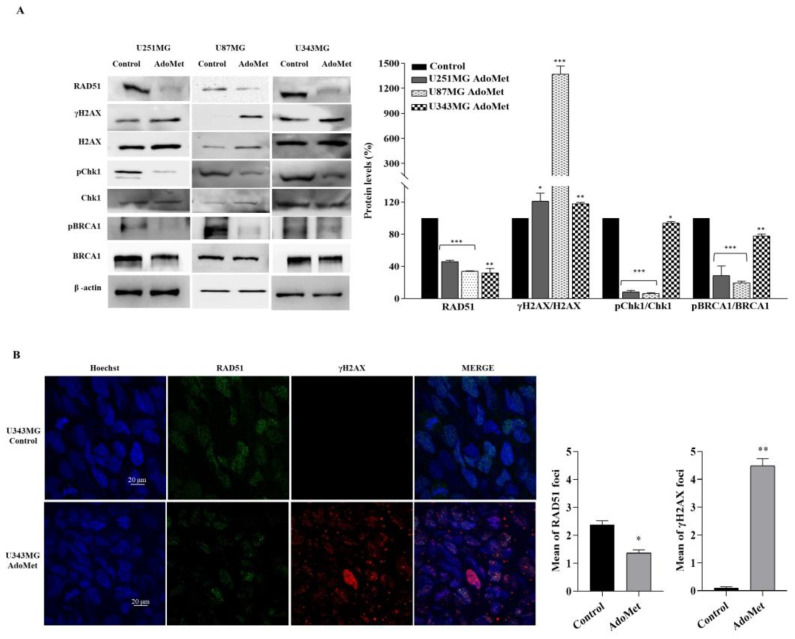
Effects of AdoMet on DNA damage response in GBM cells. (**A**) U251MG, U87MG, and U343MG cells were cultured for 72 h in medium supplemented or not (control) with AdoMet 500 μM. The levels of the main proteins involved in DNA repair and DNA damage response were then evaluated by Western blot, and the relative densitometric analyses, performed for each protein in relation to its relative housekeeping included in the Supplementary Figures S7–S9 (showing the uncropped images of Western blots), are reported as a percentage of untreated cells (100%). Representative housekeeping β-actin protein, used as a loading control, was reported. Error bars represent the SD. * *p* < 0.05, ** *p* < 0.01, *** *p* < 0.005 versus untreated cells. (**B**) Effect of AdoMet on γH2AX and RAD51 foci formation. Representative images of immunofluorescence staining for phosphorylated γH2AX (red) and RAD51 (green) in U343MG cells treated or not (control) with AdoMet. The overlapping of the two signals (merge panel) in AdoMet-treated cells evidenced the reduced expression of RAD51 and the concomitant increase in γH2AX foci. Hoechst 33258 was used for nuclear staining (blue). Scale bar: 20 μm. About 100 nuclei for each group were scored in each experiment, and a threshold of five foci per cell was considered positive and reported in the histograms as a mean of γH2AX and RAD51 foci. Values represent the means of three experiments ± SD (* *p* < 0.05, ** *p* < 0.01).

GBM is extremely resistant to therapies due to the enhanced activity of DNA repair systems which render DNA damaging treatments ineffective [42]. Homologous recombination (HR) is a major pathway for the repair of DNA double-strand break (DSB), the most harmful type of DNA lesions [43]. Tumor cells with defective HR show increased sensitivity to chemotherapeutic agents, suggesting that HR inhibition could offer great opportunities for designing targeted therapeutic strategies [44]. To investigate whether AdoMet was able to target DNA damage response in GBM, we incubated U87MG, U251MG, and U343MG cells with 500 μM AdoMet for 72 h, and then we examined via Western blot the levels of key proteins involved in HR repair (Figure 5A). We firstly analyzed RAD51 recombinase, the major catalyst of HR that provides an error-free repair of DSB during cell cycle phases S and G2 [45]. We found that AdoMet treatment lowered the expression of RAD51 in the three GBM cell lines examined compared with the untreated cells. A complex network of hierarchically ordered and reciprocally coordinated proteins are involved in the HR pathway to identify, signal, and repair DNA DSB [46]. Among them, we examined breast cancer type 1 susceptibility protein (BRCA1) and the checkpoint kinase 1 (Chk1), two important players in DNA damage response [47,48]. Western blot analysis evidenced that, following AdoMet treatment, the ratio between the phosphorylated and not phosphorylated forms of Chk1 and BRCA1 decreased with respect to the control cells, indicating that AdoMet could effectively inhibit the activation of the HR pathway, thus promoting the progression of cells with unrepaired DNA damage into mitosis.

### 2.5. AdoMet-Induced Downregulation of RAD51 Is Associated with Increased γH2AX Levels

To assess whether the AdoMet-induced downregulation of RAD51 expression was related to unrepaired damaged DNA, we analyzed the levels of the histone variant H2AX and its phosphorylated form (γ-H2AX). Notably, γH2AX is considered the most sensitive marker for DNA DSB and is required for the formation of DNA damage foci through the recruitment of DNA repair proteins at the sites containing damaged chromatin [49]. As reported in the literature, the quantification of γH2AX levels relative to total H2AX expression could provide a normalized value representative of the amount of DNA damage in cancer cells [50]. Accordingly, we found that in AdoMet-treated U87MG, U251MG, and U343MG cells, the inhibition of HR repair was associated with the increased γH2AX/H2AX ratio compared to the control (Figure 5A) indicating the presence of damaged DNA. Confirming the Western blot results, the immunofluorescence staining of U343MG cells showed that the signal intensity of γH2AX, which was almost undetectable in untreated cells, became evident in the nuclei of AdoMet-treated cells, revealing the formation of DNA damage foci (Figure 5B). Concomitantly, the signal coming from RAD51 decreased, evidencing a defective DNA repair (Figure 5B). These findings indicated that in GBM cells, AdoMet was able to keep DNA in a damaged state through inhibiting its repair.

### 2.6. AdoMet Promoted Mitotic Catastrophe

Mitotic catastrophe is considered an onco-suppressive mechanism that directs cells that are unable to complete mitosis to an irreversible antiproliferative fate of a delayed mitosis-related death or permanent cell cycle arrest with subsequent senescence [51,52,53].

**Figure 6:**
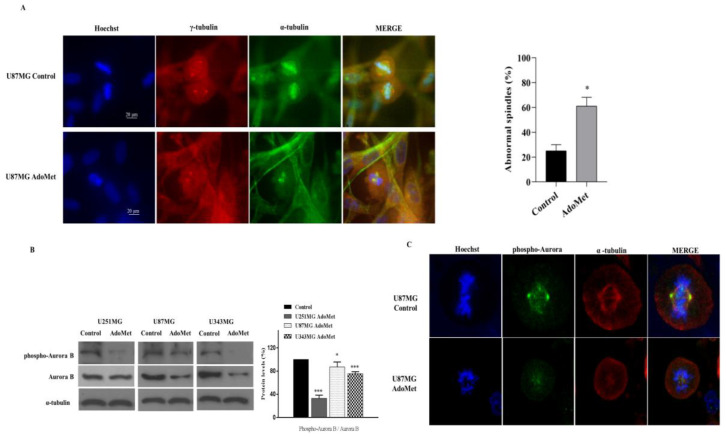
Effect of AdoMet on mitotic microtubule and spindle organization and Aurora B kinase inhibition in GBM cells. (**A**) Representative immunofluorescence images of U87MG cells treated or not (control) with 500 μM AdoMet. Mitotic tubulins were immunostained with antibodies anti γ-tubulin (red) and α-tubulin (green). Hoechst 33258 was used for nuclear staining (blue). Images are representative of three independent experiments. Scale bar 20 μm. As shown, AdoMet-treated cells exhibited missegregated chromosomes, decondensed chromatin, and the absence of the spindle (α-tubulin, green) and centrosome (γ-tubulin, red), indicative of mitotic catastrophe. Histogram shows the percentages of abnormal spindle formation from three separate experiments (represented as mean ± S.D.). Per experiment, 100 mitotic cells were counted from the control samples and the AdoMet-treated samples. AdoMet-treated samples showed mitotic cells that contained significantly more abnormal spindles than the control samples (* *p* < 0.05). (**B**) Aurora B and phospho-Aurora B levels were evaluated by Western blot, and the relative densitometric analyses performed for each protein in relation to its relative housekeeping protein were reported as a percentage of untreated cells (100%). Uncropped images of Western blots and their housekeeping proteins are reported in Figure S10. Error bars represent the SD. * *p* < 0.05, *** *p* < 0.005 versus untreated cells. (**C**) Representative confocal images showing the localization of phospho-Aurora during cell division. U87MG cells treated or not (control) with 500 μM AdoMet for 72 h were stained with antibodies anti-pospho-Aurora (green) and anti α-tubulin (red). Hoechst 33258 was used for nuclear staining. As shown, in AdoMet-treated cells, the fluorescence signal of phospho-Aurora appears largely diffused without concentrated localizing to the kinetochore with altered mitotic spindles, deranged chromatin condensation (blue), and microtubule disorganization (red). Scale bar: 5 μm, zoom factor 1.4.

The ability of AdoMet to arrest cell cycle in S and G2/M phases and to maintain DNA in a damaged state by inhibiting DDR prompted us to investigate the effects of the sulfonium compound on mitotic catastrophe-related cell death, an antitumor role not yet reported so far for AdoMet. Mitotic catastrophe is described as an aberrant form of mitosis associated with the presence of cells with an abnormal configuration and spatial rearrangements of chromosomes. These features can be used as morphological markers for identification. On this basis, to obtain direct evidence of AdoMet-induced mitotic catastrophe we examined the morphology of tumor cells after AdoMet treatment by performing immunofluorescence staining of U87MG cells with α- and γ-tubulin antibodies to analyze microtubules and the mitotic spindle, while chromosomes were stained with Hoechst 33258. Interestingly, as shown by the fluorescent images reported in Figure 6A, control cells in division showed well-organized bipolar mitotic spindles with the chromosomes (blue) aligned on the equatorial plate, while the AdoMet-treated cells in division showed disorganized chromosomes (blue) and the absence of mitotic spindle (α-tubulin, green) and centrosome (γ-tubulin, red) (Figure 6A). This finding provided evidence that AdoMet induced morphological changes in U87MG cells, indicative of cells that had undergone mitotic catastrophe.

### 2.7. AdoMet Targeted the Expression, Activation, and Subcellular Localization of Aurora B

Aurora B is a mitotic serine/threonine protein kinase belonging to the Aurora kinase family whose activity is essential for the precise and accurate chromosome segregation and execution of cytokinesis [54]. To perform these critical functions, Aurora B needs to be in the active phosphorylated status, allowing its dynamic localization at different subcellular positions where it is involved in key mitotic events such as the correction of erroneous kinetochore–microtubule attachment, the activation of the mitotic spindle assembly checkpoint until accurate bipolar spindle attachment is achieved, and cytokinesis [55]. To assess whether AdoMet-induced mitotic catastrophe could be ascribed to the inhibition of Aurora B, we firstly analyzed the levels of the total protein and its phosphorylated form after treatment of U251MG, U87MG, and U343MG cells with 500 μM AdoMet for 72 h. As evidenced by Western blot analysis, both Aurora B and phospho-Aurora B were resultingly downregulated in AdoMet-treated cells compared with the control (Figure 6B). To confirm this result and to obtain more direct evidence on the effect exerted by AdoMet on Aurora B activation and function, we performed an immunofluorescent analysis with confocal microscopy to detect the expression and subcellular localization of phospho-Aurora B (Figure 6C). We found that in control cells, phospho-Aurora B (green signal) was localized mainly at the mitotic spindle poles and partly in the packed chromosomes, while in AdoMet-treated cells, the protein signal appeared weaker and largely diffused without concentrated localization at the kinetochore. Altogether, these data provided evidence indicating that AdoMet was able to target the expression and activation of Aurora B kinase, resulting in impairment of its subcellular localization and normal spindle microtubules assembly.

### 2.8. AdoMet Suppressed Cell Proliferation and Survival in Patient-Derived GBM Cells

Several research studies have demonstrated that established GBM cell lines accumulate mutations that can produce changes in cell genotypes and phenotypes compared to the original tumors from which they are derived. Conversely, it has been proposed that low-passage primary cultures may represent much more relevant models for in vitro studies [56]. Currently, primary GBM cells isolated from native body tissue represent the gold standard for in vitro drug screening. Accordingly, to provide physiological relevance to our findings and to gain insight into the ex vivo effect of AdoMet, we performed a detailed analysis of the antitumor potential of this compound in two primary cell lines, GBM3 and GBM4, derived from fresh tumoral masses of two selected GBM patients. Primary GBM cells were characterized for the wild-type IDH1/IDH2 status, which helps with prognosis, MGMT methylation status, which indicates the efficacy of current standard of care, and finally, the epigenetic subclass of the samples used, as reported in a previous paper [57].

**Figure 7:**
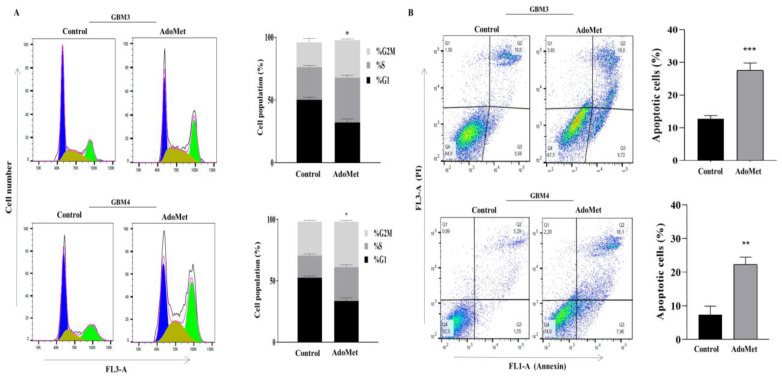
Effects of AdoMet on cell cycle distribution and apoptotic process in primary GBM cells. GBM3 and GBM4 cells were cultured for 72 h in medium supplemented or not (control) with AdoMet 500 μM, and the cell cycle (**A**) and apoptotic process (**B**) were assessed by FACS analysis. For each sample, at least 2 × 10^4^ events were analyzed. Data represent the average of three independent experiments. (**A**,**B**) Histograms show the percentage of cells in each phase of cell cycle and the percentage of apoptotic cells. Error bars depict the SDs. * *p* < 0.05, ** *p* < 0.01, *** *p* < 0.005 versus untreated cells.

We demonstrated that, in analogy to that observed in U87MG, U251MG, and U343MG cells, AdoMet induced cell cycle arrest in primary GBM cell lines, as evidenced by the increase in G2/M cell population from 19.8% to 29.9% and from 27.8% to 37%, with respect to the control, in GBM3 and in GBM4 cells (Figure 7A). We found that AdoMet was also able to induce apoptosis as detected by Annexin V/PI staining that revealed an increase in apoptotic cells of about 15% in both GBM3 and GBM4 cells (Figure 7B).

**Figure 8:**
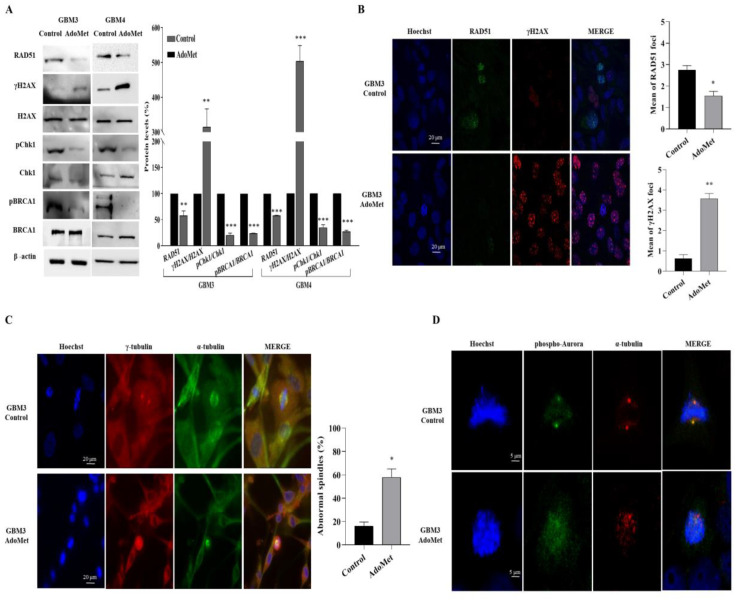
Effects of AdoMet on DNA damage response, mitotic microtubule, and spindle organization and Aurora B kinase inhibition in primary GBM cells. (**A**) GBM3 and GBM4 cells were cultured for 72 h in medium supplemented or not (control) with 500 μM AdoMet. The levels of the main proteins involved in DNA repair and DNA damage response were then evaluated by Western blot, and the relative densitometric analyses performed for each protein in relation to its relative housekeeping protein were reported as a percentage of untreated cells (100%). Uncropped images of Western blots and their housekeeping proteins are reported in Figures S11 and S12. Representative housekeeping β-actin protein, used as a loading control, was shown. Error bars represent the SD. * *p* < 0.05, ** *p* < 0.01, *** *p* < 0.005 versus untreated cells. (**B**) Representative images of immunofluorescence staining for γH2AX (red) and RAD51 (green) in GBM3 cells treated or not (control) with AdoMet. The overlapping of the two signals (merge panel) in AdoMet-treated cells highlighted the reduced expression of RAD51 and the concomitant increase in γH2AX foci. γH2AX foci and RAD51 foci were counted and plotted as histograms. Hoechst 33258 was used for nuclear staining (blue). Scale bar: 20 μm. About 100 nuclei for each group were scored in each experiment, and a threshold of 5 foci per cell was considered positive and reported in the histograms as a mean of γH2AX and RAD51 foci. Values represent the means of three experiments ± SD (* *p* < 0.05). (**C**) Representative immunofluorescence images of GBM3 cells treated or not (control) with AdoMet. Mitotic tubulins were immunostained with antibodies anti γ-tubulin (red) and α-tubulin (green). Hoechst 33258 was used for nuclear staining (blue). Images are representative of three independent experiments. Scale bar: 20 μm. Per experiment, 100 mitotic cells were counted from the control and the AdoMet-treated samples. AdoMet-treated samples showed mitotic cells that contained significantly more abnormal spindles than the control samples. (**D**) Representative confocal images showing the localization of α-tubulin (red) and phospho-Aurora (green) during cell division. Hoechst 33258 was used for nuclear staining (blue). Images are representative of three independent experiments. Scale bar: 5 μm, zoom factor: 1.4.

We then assessed the effect of AdoMet on DNA repair and DNA damage response in patient-derived GBM cells. As shown in Figure 8A, Western blot analysis evidenced, in the treated cells, a decreased level of the expression and activation of HR-relevant proteins, including RAD51, Chk1, and BRCA1, indicating that AdoMet inhibited the process of DNA repair in GBM3 and GBM4 cells. We also demonstrated that AdoMet treatment increased the γH2AX/H2AX protein ratio compared with the control. Concomitantly, as shown by immunofluorescence staining experiments, the intensity of the γH2AX signal, indicative of the formation of DNA damage foci, increased in the nuclei of AdoMet-treated cells, while the signal coming from RAD51 decreased (Figure 8B), furnishing evidence that AdoMet was able to target DNA damage response in primary GMB cells. Similar to what was previously observed in stabilized GBM cells, confocal microscopy images of AdoMet-treated GBM3 cells highlighted morphological alterations of mitotic spindle, associated with deranged chromatin condensation and microtubule disorganization (Figure 8C). Finally, AdoMet treatment resulted in a decrease in the intensity of the phospho-Aurora B signal and its delocalization from the centromeres/kinetochores (Figure 8D), confirming the ability of the sulfonium compound to inhibit Aurora B kinase and to induce mitotic catastrophe.

All together, these data demonstrated that the mechanisms operated by AdoMet to inhibit growth and survival in stabilized GBM cell lines were preserved in primary cell models that better reproduce the features of the original tumor.

## 3. Discussion

GBM is the most prevalent malignant tumor of the central nervous system whose features of heterogeneity, infiltration of surrounding brain tissue, and multidrug resistance, mainly due to the innate upregulation of DNA repair mechanisms, are responsible for the extremely poor survival rate of patients [1,2]. This points up the need for novel and improved therapeutic approaches.

In the last few years, natural products or plant-based drugs acquired much interest as a potential and effective tool to treat many pathological conditions or as alternatives to modern medicines. Recent findings have documented that natural compounds exhibit antitumor effects by interfering with the initiation, development, and progression of cancer through acting as epigenetic regulators and modulators of several tumorigenic signaling pathways [58]. Natural compounds have therefore been evaluated for their potential as agents in GBM treatment [59]. Although the beneficial effects of natural compounds are promising, their efficacy in GBM is limited by their poor bioavailability and blood–brain barrier permeability which prevent them from reaching the brain parenchyma and consequently exerting their therapeutic effects. Several strategies have been set up to overcome these obstacles, among which nanotechnologies hold great promise for the treatment of GBM [60].

Much evidence, in recent years, has highlighted AdoMet, the physiological methyl donor and epigenetic regulator, as a modulator of multiple cancer-related signaling pathways and a promising chemosensitizing agent in various cancer types [16,21,22,23,24,25,26,27,28,29,30].

In the current study, we explored the anticancer activity of AdoMet in both stabilized and primary human GBM cell lines and provided the first evidence that AdoMet was able to inhibit DNA repair and to induce mitotic catastrophe through downmodulating the expression and activity of Aurora B kinase.

The peculiar feature of natural compounds, including AdoMet, as chemotherapeutic drugs is their ability to efficiently kill tumor cells without exerting, under the same experimental conditions, any significant cytotoxic effect in normal cells. Accordingly, to evaluate the cytotoxicity of AdoMet in GBM cells and its selectivity towards tumor cells, we carried out comparative MTT assays which represent an experimental strategy generally utilized to test the selective drug cytotoxicity. We found that AdoMet selectively and efficiently caused a time- and dose-dependent inhibition of U87MG, U251MG, and U343MG cell viability without exerting any detectable cytotoxicity in non-tumorigenic NHA. A conceivable explanation for this behavior could be that although AdoMet is a general methyl donor that acts on both normal and cancer cells, the transcriptional landscape on which it acts is different, resulting in different phenotypic effects of AdoMet on cancer and normal cells.

It is interesting to note, in this regard, that AdoMet, currently used as a preventive agent for mood disorders, fibromyalgia, and joint pain, is a safe and FDA-approved dietary supplement that at pharmacological doses shows a low incidence of side-effects with an excellent record of tolerability [21,61,62]. Notably, the observation that the peripheral administration of AdoMet results in the increase in AdoMet levels in cerebrospinal fluid and the evidence that AdoMet is effective in treating a number of neurological disorders suggest that this molecule crosses the blood–brain barrier [21,63]. In vitro studies employing the immortalized rat brain endothelial cell line RBE4, an established model of the blood–brain barrier, evidenced a significant interaction of AdoMet with the adenosine carrier, strongly suggesting that AdoMet may enter the central nervous system via Na^+^-independent nucleoside carrier systems at the brain capillary endothelium [63].

The cell cycle is a complex process in which a well-ordered sequence of irreversible transitions from one phase to the next is able to allow precise and accurate DNA replication, resulting in the final generation of two identical daughter cells. In cancer, cell cycle arrest, particularly at G2/M, might be a useful strategy to prevent cell proliferation or to trigger apoptotic cell death, and it has been reported that many anticancer agents reduce malignant growth by arresting cell cycles at G1/S or G2/M phases [64].

The Cdc25 family encompasses three highly conserved members of dual-specificity phosphatases that through removing inhibitory phosphate groups from cyclin-dependent kinases regulate cell cycle progression in the S phase and mitosis. Specifically, Cdc25C causes the full activation of CDK1 at mitotic entry [41]. Following DNA damage, the inactivation of Cdc25C by phosphorylation functions as a checkpoint regulatory mechanism that prevents the cells from entering mitosis in order to allow DNA damage repair or alternatively to initiate cell apoptosis [65]. Cdc25 phosphatase is therefore considered an attractive target for anticancer drug discovery [66]. It has to be pointed out that Cdc25 inactivation is a major mechanism of p53-independent checkpoints and could therefore play a particularly relevant role for DNA damage response in cancer cells characterized by high levels of mutant p53, such as GBM [67].

Interestingly, we found that AdoMet induced cell cycle arrest in GBM cells and that AdoMet treatment resulted in the increase in phosho-cdc25c protein, suggesting that AdoMet could retard GBM cell proliferation via phospho-cdc25c upregulation to arrest cell cycle progression at the G2/M phase.

The ability of GBM cells to escape from programmed cell death due to the intrinsic dysregulation of mechanisms involved in the control of cell cycles and apoptosis not only promotes tumorigenesis but also plays a critical role in providing resistance to current treatments. In this context, the identification of bioactive molecules able to enhance apoptosis without causing significant toxic side effects represents a major clinical challenge. Consistent with this view, our data highlighted the ability of AdoMet to trigger apoptotic cell death in GBM cells as evidenced by the activation of caspase 3 and the cleavage of PARP-1.

DSBs are the most damaging DNA lesions that, if not repaired, can cause insertions, deletions, or chromosomal rearrangements. The detection, signaling, and repair of DSBs involve a complex network of cellular events collectively referred to as DNA damage response. A large fraction of the DSBs is repaired by the HR system which exchanges equivalent regions of DNA between homologous or sister chromosomes and therefore is most active in late-S/G2 phases of the cell cycle when DNA has been replicated and each cell disposes two copies of each DNA strand. HR provides an error-free adjustment of DSBs, contributing to the safeguarding of genomic integrity and ensuring a high-fidelity transmission of genetic information [43].

RAD51, BRCA1, and Chk1 are key proteins involved in the HR pathway. RAD51 is an ATPase that forms a nucleoprotein strand on single-stranded DNA and has the function of finding and invading homologous DNA sequences to enable accurate and timely DNA repair [68,69]. Notably, RAD51 is expressed at high levels in GBM and is associated with a poor outcome and reduced response to cytotoxic treatments such as chemotherapy and radiotherapy [70]. Thus, downregulating RAD51 expression and/or interfering with its function could be of great therapeutic value [47,71]. BRCA1 is a large multi-domain protein that maintains genome stability by promoting HR-mediated DSB repair [47,71] and is required for the efficient loading of RAD51 at DNA damage sites and its stabilization for HR processes [72]. Chk1 is an important transducer of DNA damage signals, and its activation leads to cell cycle arrest at S and G2 checkpoints to promote DNA repair before cell division [48]. Chk1 is activated by phosphorylation in response to DNA damage and in turn phosphorylates a variety of intracellular substrate proteins, including the recombinase RAD51, allowing its recruitment to DNA damage sites [73]. Notably, we found that (i) AdoMet was able to downregulate the expression of RAD51 and the activation of BRCA1 and Chk1, as evidenced by the decreased protein ratio between the phosphorylated and unphosphorylated forms of these proteins; (ii) as highlighted by the increase in the γ-H2AX/H2AX ratio, AdoMet treatment resulted in the activation of H2AX, the highly conserved histone H2AX variant that in response to DSBs becomes phosphorylated to form γ-H2AX and participates to the recruitment of DDR-related factors to the lesion sites, further propagating the DNA damage signal; and (iii) AdoMet treatment resulted in the decrease in RAD51 foci formation associated with the increase in the immunofluorescence signal of γ-H2AX foci, indicating the AdoMet induced downregulation of DDR with the consequent increase in damaged DNA. All together, these findings provided the first evidence of the ability of AdoMet to exert anticancer activity in GBM cells through inhibiting HR repair, thus maintaining DNA in a persistent damaged state, a condition that can lead to different death-associated consequences, including mitotic catastrophe [74]. Accordingly, it has been reported that in HeLa cells after irradiation, mitotic DNA damage delays mitotic exit and prevents cytokinesis, resulting in mitotic catastrophe [75]. Since the standard therapy for GBM consists of a combinatory treatment of ionizing radiation and alkylating drugs which both damage DNA, the use of natural molecules, such as AdoMet, which are able to target DDR could represent a promising beneficial adjuvant strategy to sensitize tumor cells to treatment through triggering mitotic catastrophe-induced cell death.

To protect the integrity of the genome during cell cycle progression, in response to genotoxic stress, the cells activate three major checkpoints; the first occurring near the end of the G1 phase, the second at the G2/M phase transition, and the third, also called spindle assembly checkpoint, at the metaphase-to-anaphase transition. This temporally and spatially controlled checkpoint activation results in the arrest of cell cycle and in the induction of DNA repair processes in order to prevent the damaged DNA from being duplicated or passed on to the next generation. In cancer, several reasons such as the dysregulation of the G2 checkpoint responsible for the premature transition of cells into mitosis before DNA has been completely repaired; the dysfunction of mitotic apparatus and feedback control systems that manage cell division; the utilization of antimitotic drugs targeting microtubules; and/or compounds interfering with proper spindle formation, chromosome segregation, and mitotic exit can lead to mitotic catastrophe and subsequent mitotic catastrophe-induced cell death [51]. Mitotic catastrophe could be therefore considered an onco-suppressive mechanism, and promoting mitotic catastrophe could be a promising strategy in cancer treatment. Accordingly, drugs inducing mitotic catastrophe have been recently explored as a new efficient alternative to the existing treatment in GBM [53]. In this context, the ability of AdoMet to promote mitotic catastrophe in GBM cells, as suggested by the morphological changes typical of this process induced by AdoMet treatment, highlighted the potential of this compound for drug development against GBM.

Aurora kinases, a closely related family of serine/threonine kinases, including Aurora A, B, and C are highly conserved mitotic kinases that act synergistically with several other proteins to control chromosome alignment and its equal distribution to daughter cells in mitosis and meiosis [55]. The deregulation of Aurora kinase genes has been reported in GBM, and the development of small molecules targeting these proteins is considered a promising strategy for the treatment of this tumor [76].

Due to its deregulation and overexpression in various tumors, Aurora B has emerged as an important therapeutic target. Small-molecule inhibitors of Aurora B have been reported to suppress the spindle assembly checkpoint causing premature mitotic exit and consequent chromosome missegregation, cytokinesis failure, and nuclear fragmentation resulting in mitotic catastrophe and cell death [77]. Notably, we found that AdoMet was able to downregulate the expression and activation of Aurora B in GBM cells and to impair its proper localization, an important prerequisite for the kinase to properly perform its functions.

## 4. Materials and Methods

### 4.1. Materials

Propidium iodide (PI) (Catalog No. P4864), 3-(4,5-dimethylthiazol-2-yl)-2,5-diphenyltetrazolium bromide (MTT) (Catalog No M2003), and radioimmunoprecipitation assay buffer (RIPA buffer) (Catalog No. R0278) were obtained from Sigma-Aldrich (St. Louis, MO, USA). Bovine serum albumin (BSA), Dulbecco’s modified Eagle’s medium (DMEM), DMEM/Ham’s F-12, fetal bovine serum (FBS), phosphate-buffered saline (PBS), and trypsin–EDTA were acquired from Gibco (Thermo Fisher Scientific, Monza, Italy). Non-essential amino acids, astrocyte basal medium (ABMTM), and astrocyte growth medium (AGMTM) were bought from Lonza (Rome, Italy). AdoMet (Catalog No. B9003S) was purchased from New England Biolabs (dissolved in 5 mM H2SO4 and 10% ethanol and filtered). The annexin V-fluorescein isothiocyanate (V-FITC) apoptosis detection kit (Catalog No. 556547) was obtained from eBioscience (San Diego, CA, USA). Protein analysis dye reagent concentrate (Catalog No. 5000006) and Trans-Blot Turbo (Catalog No. 1704158) were bought from Bio-Rad (Hercules, CA, USA). Monoclonal antibodies (mAb) to poly(ADP ribose)polymerase (PARP-1) (46D11 #9532), p21 (12D1 #2947), cyclin D1 (92G2 #2978), CDK4 (#12790), cyclin E1 (HE12 #4129), cyclin A2 (BF683 #4656), cdc25c (#4688), RAD51 (D4B10), pBRCA1 (Ser1524 #9009), pChk1 (Ser345 #2341), phospho-Rb (Ser795 #9301), phospho-Aurora B (Thr232) (#2914), β-actin (8H10D10 #3700), α-tubulin (11H10 #2125 and DM1A #3873), and polyclonal antibodies (polyAb) to caspase 3 (#9662), cyclin B1 (#4138), and phospho-cdc25c (#9528) were acquired from Cell Signaling Technology (Danvers, MA, USA). Monoclonal antibodies (mAb) to Chk1 (sc-8408), BRCA1 (sc-1021), histone H2AX (sc-517336), p-Histone H2AX (sc-517348) and β-actin (sc-47778) were acquired from Santa Cruz Biotechnology (Dallas, TX, USA). PolyAb to Aurora B (36-5200), Rb (A302-432A), and Hoechst 33258 pentahydrate (bis-Benzimide) (Catalog No. H3569) for nuclei staining were purchased from Invitrogen (Thermo Fisher Scientific, Monza, Italy). Monoclonal antibody to γ-tubulin (t6557) was obtained from Sigma-Aldrich (St. Louis, MO, USA). Horseradish peroxidase (HRP)-conjugated goat anti-mouse (GxMu-003-DHRPX) and HRP-conjugated goat anti-rabbit (GtxRb-003-DHRPX) secondary antibodies were purchased from Jackson ImmunoResearch Laboratories Inc. (Raleigh, NC, USA). All buffers and solutions were prepared with ultrahigh quality water. All reagents were of the purest commercial grade.

### 4.2. Cell Cultures

U87MG of unknown origin (https://web.expasy.org/cellosaurus/CVCL_0022, (accessed on 28 August 2020) and U251MG stabilized cell lines were obtained from Elabscience (Houston, TX, USA; Catalog No. EP-CL-0238 and Catalog No. EP-CL-0237, respectively). The authentication statement used was the genotyping of STR and amelogenin locus of the U87MG cell line. U343MG stabilized cell line was purchased from CLS Cell Lines Service GmbH (Eppelheim, Germany, Catalog No. 300365). All cell lines were cultured in tissue culture dishes (Corning, NY, USA), in DMEM supplemented with 10% heat inactivated FBS, 1% non-essential amino acids, 1% sodium pyruvate, 1% l-glutamine, and 0.1% PlasmocinTM prophylactic (InvivoGen, San Diego, CA, USA), and maintained at 37 °C in a humidified atmosphere containing 5% CO_2_. Normal human astrocytes (NHA) (Lonza, Rome, Italy, Catalog No. CC-25659) were cultured in ABMTM supplemented with AGMTM Single Quots Kit. Glioblastoma primary cell lines GBM3 and GBM4 were cultured in DMEM/Ham’s F-12 supplemented with 15% heat in activated FBS, 2% L-glutamine, 1% sodium pyruvate, 1% non-essential amino acids, and 1.5% D-glucose and grown under the same conditions. In the section “Preparation of GBM primary cell lines”, we describe the protocol for preparing and culturing the primary cell lines used in this work.

### 4.3. Preparation of GBM Primary Cell Lines

Primary cultures were set up from fresh tumor resections obtained after surgery at the Neurosurgery Service of “Antonio Cardarelli” Medical Hospital (Naples, Italy). Briefly, the tumor tissues were minced using the gentleMACS™ Dissociator following the instructions contained in the human tumor dissociation kit, (MiltenyiBiotec, Cologne, Germany. Cod#130-095-929), strained through a MACS SmartStrainer (mesh size 70 μm), and then cultured in DMEM/Ham’s F-12 supplemented as described in the “Cell Cultures” section. Tissue samples were collected according to the ethical standards of the Institutional Committee (DEL. N°897 13 August 2020). Clinical diagnosis and molecular characterization of tumor specimens were performed on a second sample that originated from each patient, following the same procedure as explained before [57].

### 4.4. Cell Viability Assay

The effect of AdoMet on cell viability was assessed by an MTT assay according to the manufacturer’s instruction. Briefly, U87MG, U251MG, U343MG, and NHA cells were plated in serum-enriched media in 96-well plates at the proper density and treated with 10% FBS fresh medium containing increasing concentrations of AdoMet (from 72 to 1000 μM) for 24, 48, and 72 h. The incubation medium was then removed, and MTT solution was added in PBS to a final concentration of 0.5 mg/mL. The cells were then incubated at 37 °C for 4 h, and the MTT-formazan crystals were solubilized in 1N isopropanol/hydrochloric acid 10% solution at 37 °C on a shaker for 20 min. The absorbance values of the solution in each well were detected at 570 nm by using a Bio-Rad IMark microplate reader (Bio-Rad Laboratories, Milan, Italy). All experiments were performed in quadruplicate. Cell viability was expressed as the percentage of absorbance values of treated samples compared to the control (100%). The IC50 values were calculated by using linear regression analysis.

### 4.5. Flow Cytometry Analysis of Cell Cycle

GBM cells were seeded in 6-well plates at a density of 10 × 10^4^ cells/well and treated with 500 μM AdoMet. After 72 h, the cells were recovered with trypsin–EDTA, washed twice with PBS, and incubated with nuclei-PI staining solution (50 μg/mL PI, 0.1% sodium citrate, 25 μg/mL RNase A, 0.1% triton in PBS) for 1 h at 4 °C in the dark [78]. Cell cycle distribution was measured by flow cytometry using a BD FACS Aria III (Becton & Dickinson, Mountain View, CA, USA). To evaluate cell cycle progression, PI fluorescence was detected as FL3-A (linear scale) using ModFIT LT RRID:SCR_016106 software (Verity Software House, Topsham, ME, USA). For each sample, 20,000 events were analyzed in at least three separate experiments, giving a standard deviation (SD) < 5%.

### 4.6. Flow Cytometry Analysis of Apoptosis

Apoptosis was evaluated by flow cytometry by using Annexin V-FITC in conjunction with a vital dye PI to distinguish apoptotic (Annexin V-FITC-positive, PI positive) from necrotic (Annexin V-FITC negative, PI positive) cells. GBM cells were seeded in 6-well plates at a density of 10 × 10^4^ cells/well and incubated in the presence of AdoMet 500 μM. After 72 h, cells were harvested by incubation with trypsin–EDTA, washed with PBS twice, and collected by centrifugation. The cells were then resuspended in 200 μL of Binding Buffer 1X and incubated with 5 μL Annexin V-FITC and 10 μL PI (20 μg/mL) for 30 min at room temperature, as recommended by the manufacturers. The determination of viable cells and early apoptotic, late apoptotic, and necrotic cells was carried out with a BD FACS Aria III (Becton & Dickinson, Mountain View, CA, USA). For each sample, 20,000 events were recorded. Analysis was carried out by triplicate determination on at least three separate experiments.

### 4.7. Protein Extraction and Western Blot Analysis

GBM cells were cultured in 6-well plates at a density of 10 × 10^4^ cells/well incubated in the presence of AdoMet 500 μM for 72 h and then processed for Western blot analysis. Briefly, after treatment, the cells were harvested, lysed on ice for 30 min, centrifuged at 18,000× *g* in an Eppendorf microcentrifuge for 30 min at 4 °C, and the supernatant was collected. Protein concentration was determined and compared with the BSA standard curve as reported in [79]. Equal amounts of sample proteins were separated by sodium dodecyl sulfate–polyacrylamide gel electrophoresis (SDS-PAGE) and electrotransferred onto nitrocellulose membranes by Trans-Blot Turbo (BIO-RAD). The membranes were washed in 10 mM Tris-HCl, pH 8.0, 150 mM NaCl, and 0.05% Tween 20 (TBST), blocked with TBST supplemented with 5% nonfat dry milk, and incubated first with specific primary antibodies overnight at 4 °C in TBST and 5% non-fat dry milk and then with HRP-conjugated secondary antibodies. All primary antibodies were used at a dilution of 1:1000 and 1:500; all secondary antibodies were used at a dilution of 1:5000. The immunoblots were then developed using enhanced chemiluminescence detection reagents ECL (Cyanagen, Bologna, Italy) and exposed to X-ray film or detected using a Bio-Rad ChemiDoc MP image sensor. All films were scanned and analyzed with Image J software 1.48 (National Institutes of Health, Bethesda, MD, USA).

### 4.8. Immunofluorescence Analysis

To perform immunofluorescence analysis, 2 × 10^4^ cells/cm^2^ of GBM cells were grown on glass cover slips placed in 24-well culture plates. After treatment with AdoMet 500 µM for 72 h, cells were fixed with 4% parformaldehyde, permeabilized with 0.2% Triton X-100, and blocked using PBS-BSA 0.4%. Cells were incubated with specific primary antibodies (anti-RAD51, anti-*p*-Histone-H2AX, anti-α-tubulin, anti-γ-tubulin) at a dilution of 1:200 overnight at 4 °C. Cells were then rinsed three times with PBS 1x and hybridized with a secondary antibody coupled to Alexa Fluor 488 (Jackson ImmunoResearch, Cambridge, UK) or Dylight 594 (Abcam, Cambridge, UK) for 1 h at room temperature. Cellular nuclei were counterstained using 2.5 µg/mL Hoechst-33258 for ~5 min. Coverslips were then mounted on glass slides. The images were acquired using the THUNDER Imaging system (Leica Microsystems Srl, Buccinasco, Italy). The quantification of γH2AX foci and RAD51 foci was performed by using Image J software 1.48 National Institutes of Health, Bethesda, MD, USA). Approximately 100 nuclei for each treatment group were scored in each experiment, and a threshold of 5 foci per cell was considered positive.

### 4.9. Confocal Laser Scanning Microscopy

GBM cells 2 × 10^4^ cells/cm^2^ were grown on glass cover slips placed in 24-well culture plates. After treatment with AdoMet 500 µM for 72 h, cells were fixed with 4% paraformaldehyde, permeabilized with 0.2% Triton X-100, and blocked using PBS-BSA 0.4%. Cells were incubated with specific primary antibodies (anti-α-tubulin and anti-p-Aurora B) at a dilution of 1:200 overnight at 4 °C. Cells were then rinsed three times with PBS 1x and hybridized with a secondary antibody coupled to Alexa Fluor 488 (Jackson ImmunoResearch, Cambridge, UK) or Dylight 594 (Abcam, Cambridge, UK) for 1h at room temperature. Cellular nuclei were counterstained using 2.5 µg/mL Hoechst-33258 for ~5 min. Coverslips were then mounted on glass slides. Microscopy images were obtained using a Zeiss LSM 700 laser scanning confocal microscope equipped with a plan apochromat 63× (NA 1.4) oil immersion objective. About 100 mitotic cells were counted from the control samples and the AdoMet-treated samples.

### 4.10. Statistical Analysis

Statistical analysis was carried out with the GraphPad Prism 7.0 software for Windows (GraphPad Software, Inc., San Diego, CA, USA). Data are expressed as mean ± standard deviation (SD) and analyzed for statistical significance using the two-tailed Student’s *t*-test. For multiple comparisons, ANOVA analysis was used, followed by Bonferroni correction. The *p*-values * *p* < 0.05, ** *p* < 0.01, *** *p* < 0.005, and **** *p* < 0.001 were considered statistically significant. All experiments were repeated at least 3 times and performed in triplicate.

## 5. Conclusions

**Figure 9:**
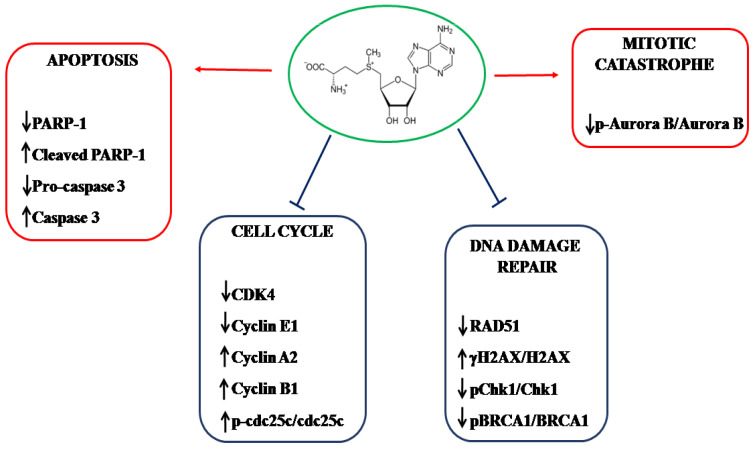
Schematic diagram summarizing the anticancer activities of AdoMet in GBM cells and the proposed molecular targets.

New advances in anticancer drug discovery using natural compounds have been made in the last few years, and AdoMet, the physiological methyl donor, has emerged as a promising molecule capable of acting as a selective cytotoxic agent leading to tumor cell death. The present study demonstrated, for the first time, that AdoMet exhibited antitumoral effects in both stabilized and primary GBM cell lines and provided experimental evidence on the underlying mechanisms. As schematically represented in Figure 9 where the anticancer activities of AdoMet and its molecular targets are summarized, AdoMet was able to kill tumor cells through inhibiting DNA repair, cell cycle progression, and Aurora B kinase expression and activity, ultimately causing cell death following the activation of apoptosis and mitotic catastrophe. The ability of AdoMet to affect cancer cells at multiple levels and thus potentially circumvent the development of resistance, together with its capability to kill tumor cells while minimally affecting normal cells, highlights the great potential of this natural compound, allowing us to propose AdoMet as a promising target for further investigations finalized to the design of innovative adjuvant therapies in GBM treatments.

## Data Availability

The datasets generated during and/or analyzed during the current study are available from the corresponding author on reasonable request. The datasets generated during the Western blot analysis are available in the Supplementary Materials.

## Supplementary Materials

The following supporting information can be downloaded at: https://www.mdpi.com/article/10.3390/molecules29081708/s1, Figure S1. Effect of AdoMet on cell cycle in U251MG cell line; Figure S2: Effect of AdoMet on cell cycle in U87MG cell line; Figure S3: Effect of AdoMet on cell cycle in U343MG cell line; Figure S4: Effect of AdoMet on apoptosis in U251MG cell line; Figure S5: Effect of AdoMet on apoptosis in U87MG cell line; Figure S6: Effect of AdoMet on apoptosis in U343MG cell line; Figure S7: Effect of AdoMet on DNA repair in U251MG cell line; Figure S8: Effect of AdoMet on DNA repair in U87MG cell line; Figure S9: Effect of AdoMet on DNA repair in U343MG cell line; Figure S10: Effect of AdoMet on phospho-AuroraB and Aurora B in U251MG, U87MG, and U343MG cell lines; Figure S11: Effect of AdoMet on DNA repair in GBM3 primary cell line; Figure S12: Effect of AdoMet on DNA repair in GBM4 primary cell line.
