# Supplementary material for: Antitumoral Activity of the Universal Methyl Donor S-Adenosylmethionine in Glioblastoma Cells
Source: Molecules. 2024 Apr 10;29(8):1708. doi: 10.3390/molecules29081708 (PMC11052366; doi:10.3390/molecules29081708)
Supplement: Supplementary file 1 [file molecules-29-01708-s001.zip › molecules-2924653-supplementary.pdf]

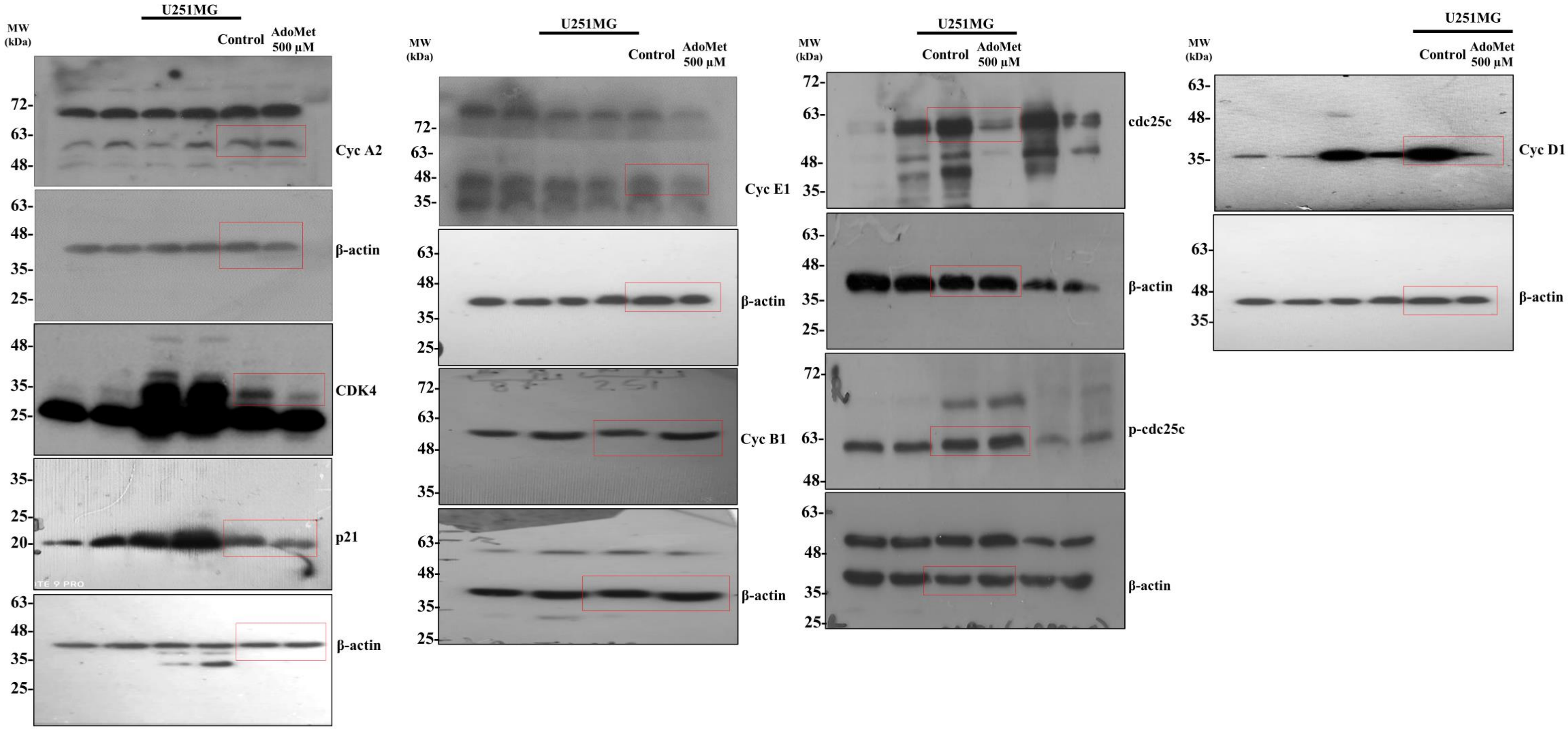

Figure S1: Effect of AdoMet on cell cycle in U251MG cells.

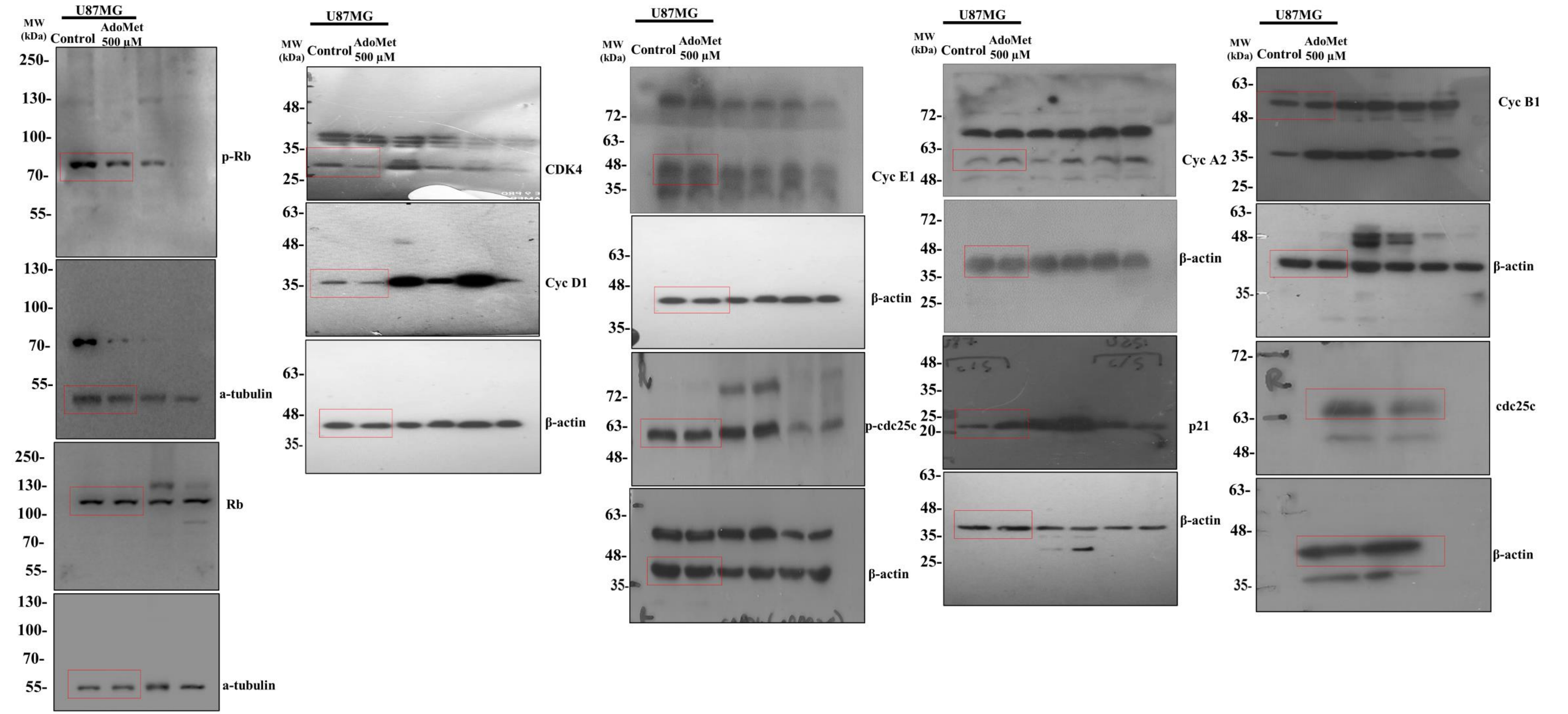

Figure S2: Effect of AdoMet on cell cycle in U87MG cells.

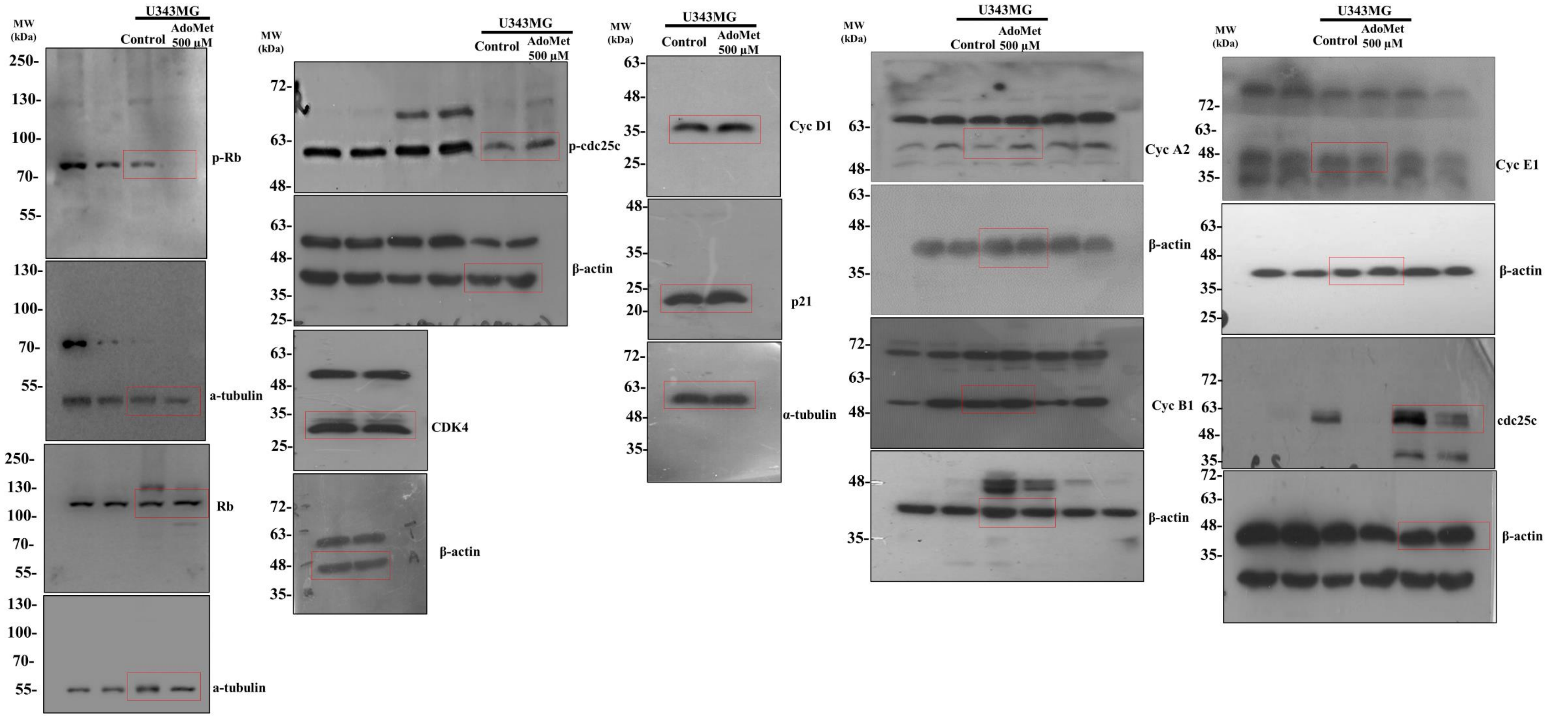

Figure S3: Effect of AdoMet on cell cycle in U343MG cells.

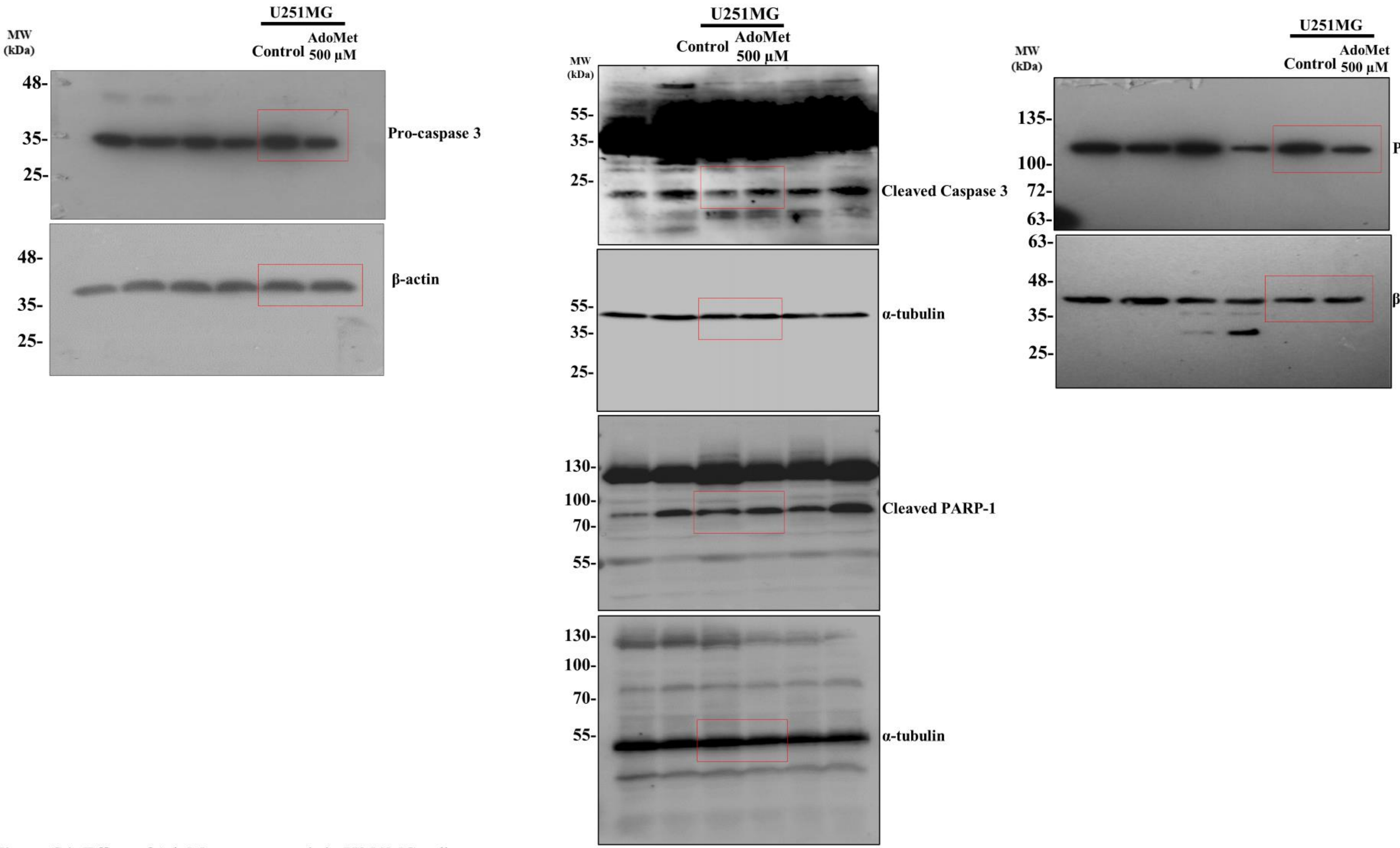

Figure S4: Effect of AdoMet on apoptosis in U251MG cells.

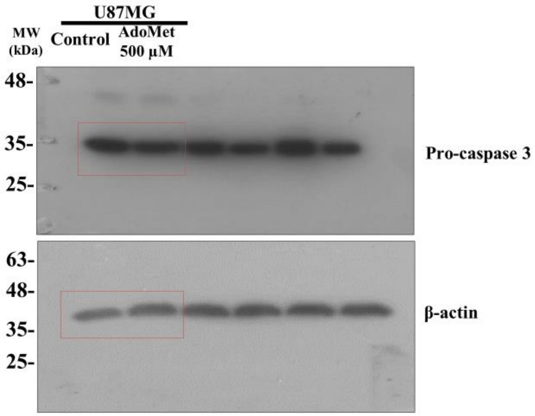

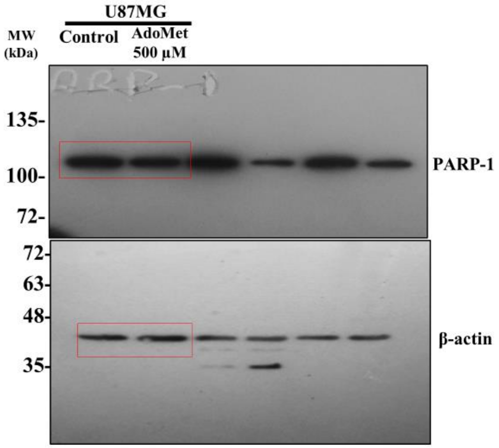

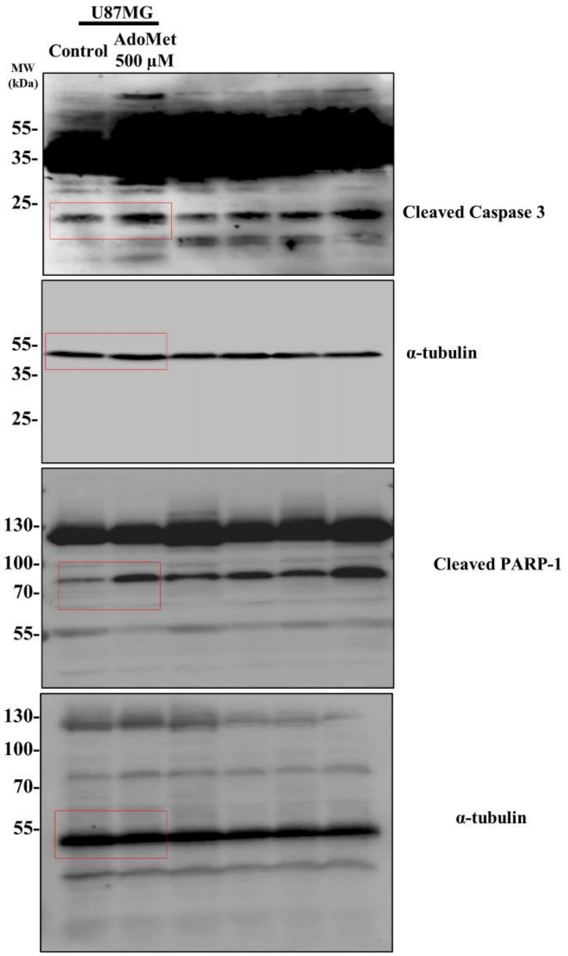

Figure S5: Effect of AdoMet on apoptosis in U87MG cells.

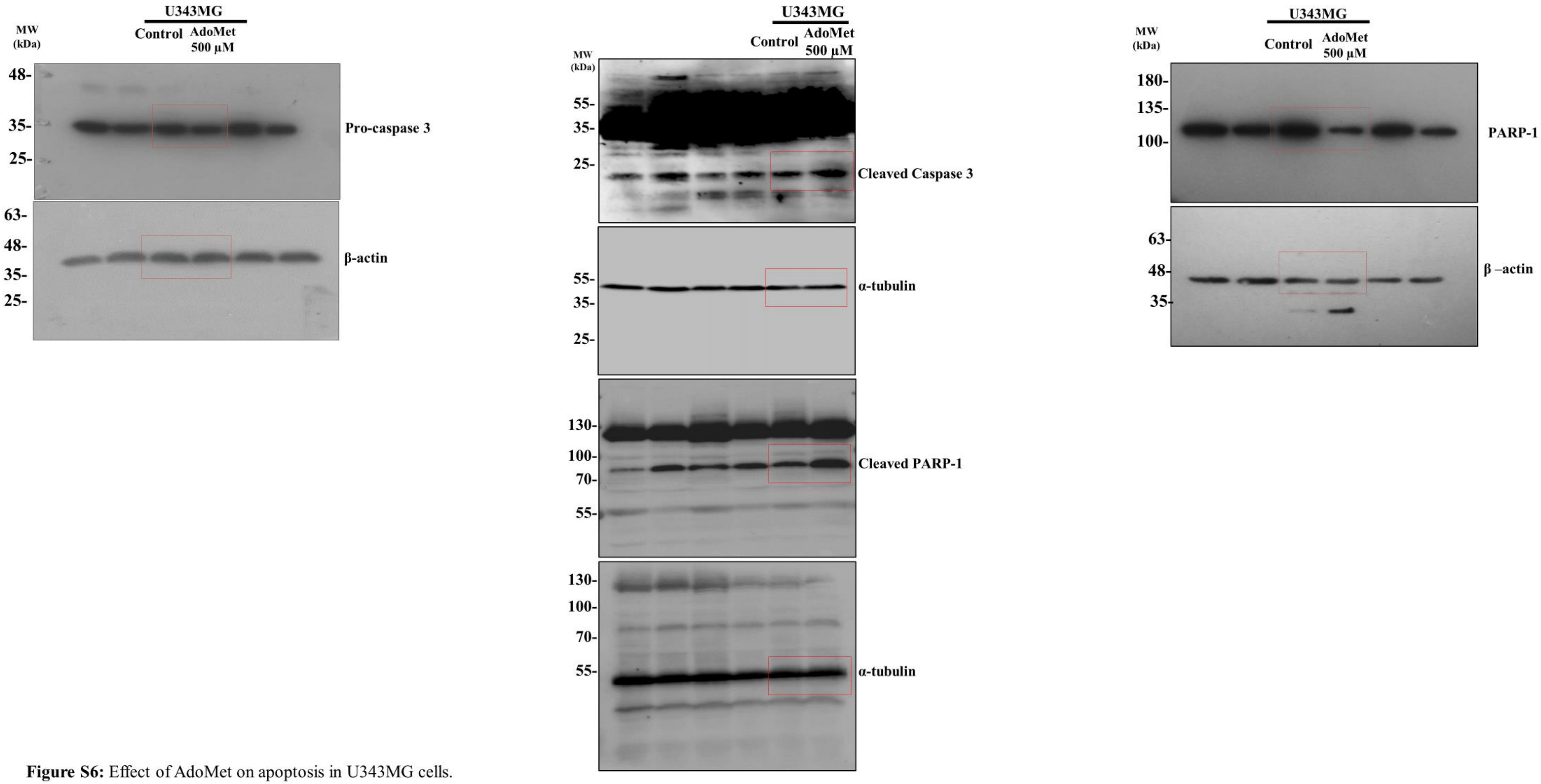

Figure S6: Effect of AdoMet on apoptosis in U343MG cells.

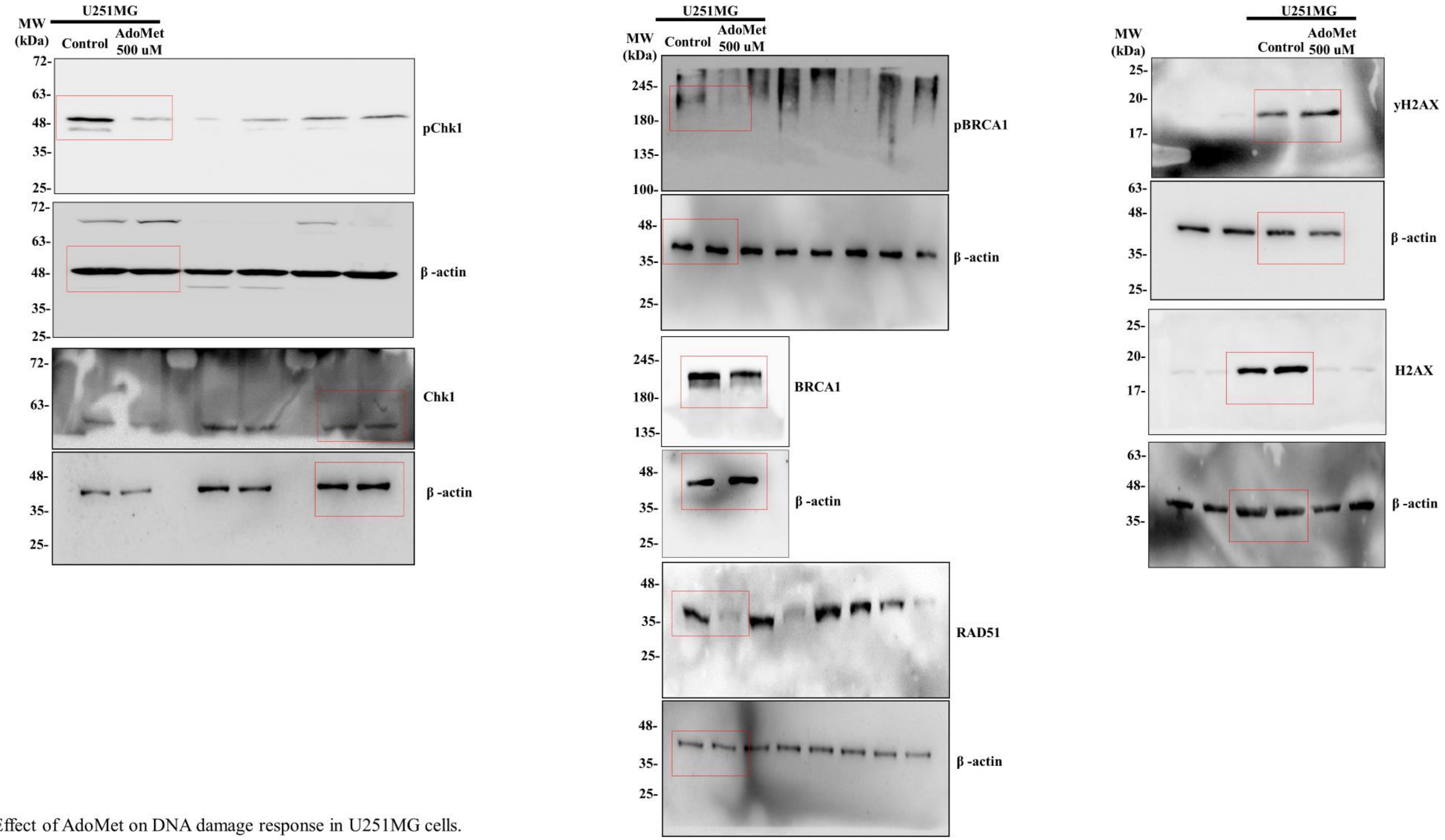

Figure S7: Effect of AdoMet on DNA damage response in U251MG cells.

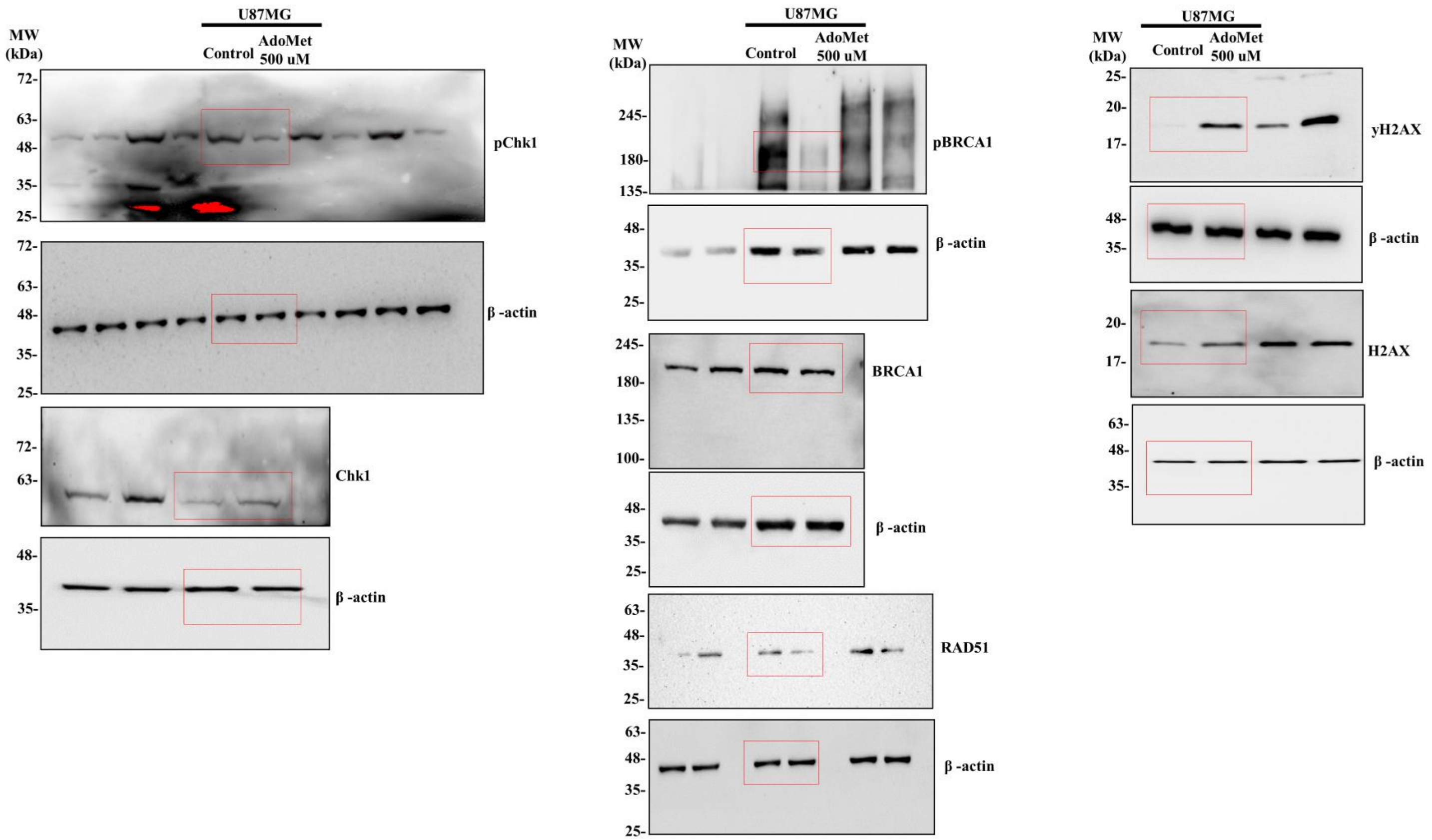

Figure S8: Effect of AdoMet on DNA repair in U87MG cells.

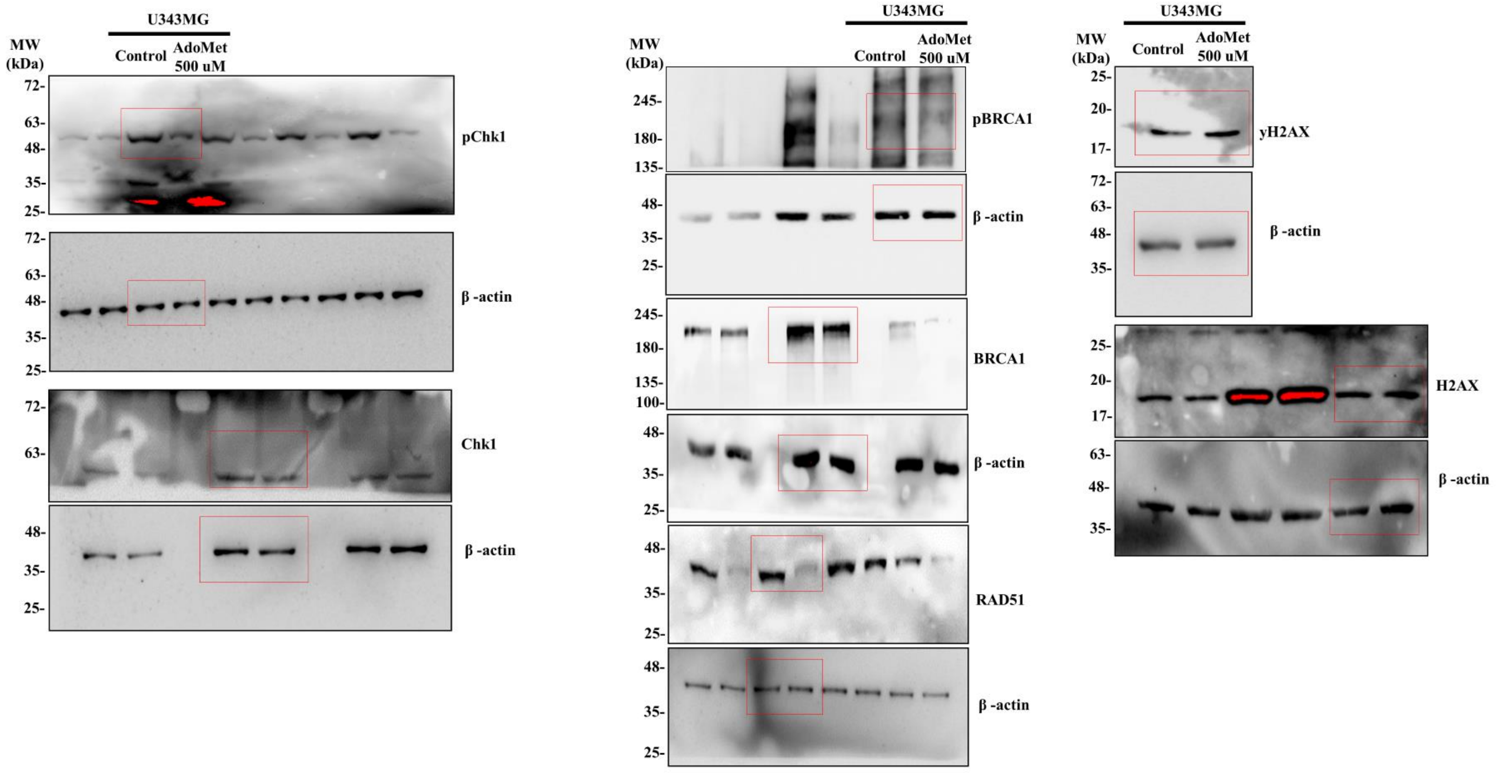

Figure S9: Effect of AdoMet on DNA repair in U343MG cells.

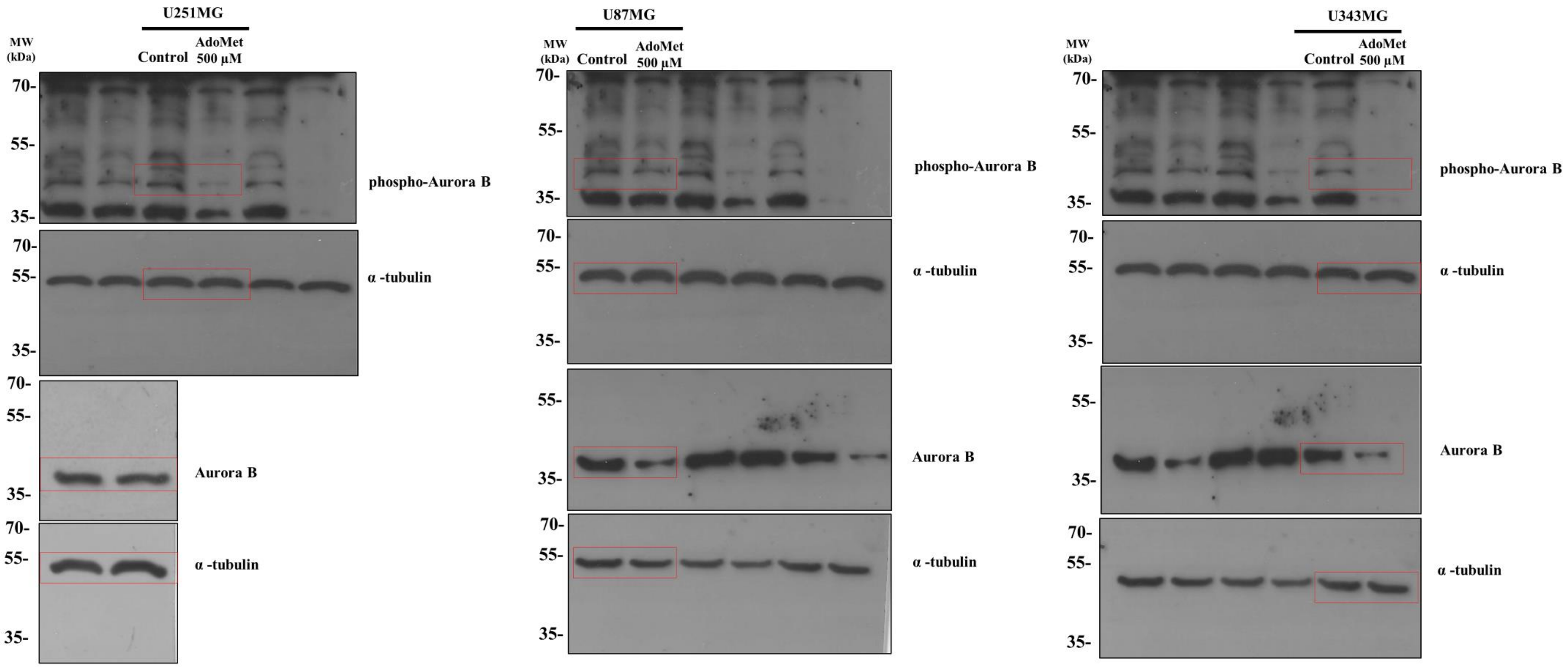

Figure S10: Effect of AdoMet on phospho-AuroraB and Aurora B in U251MG, U87MG, and U343MG cell lines.

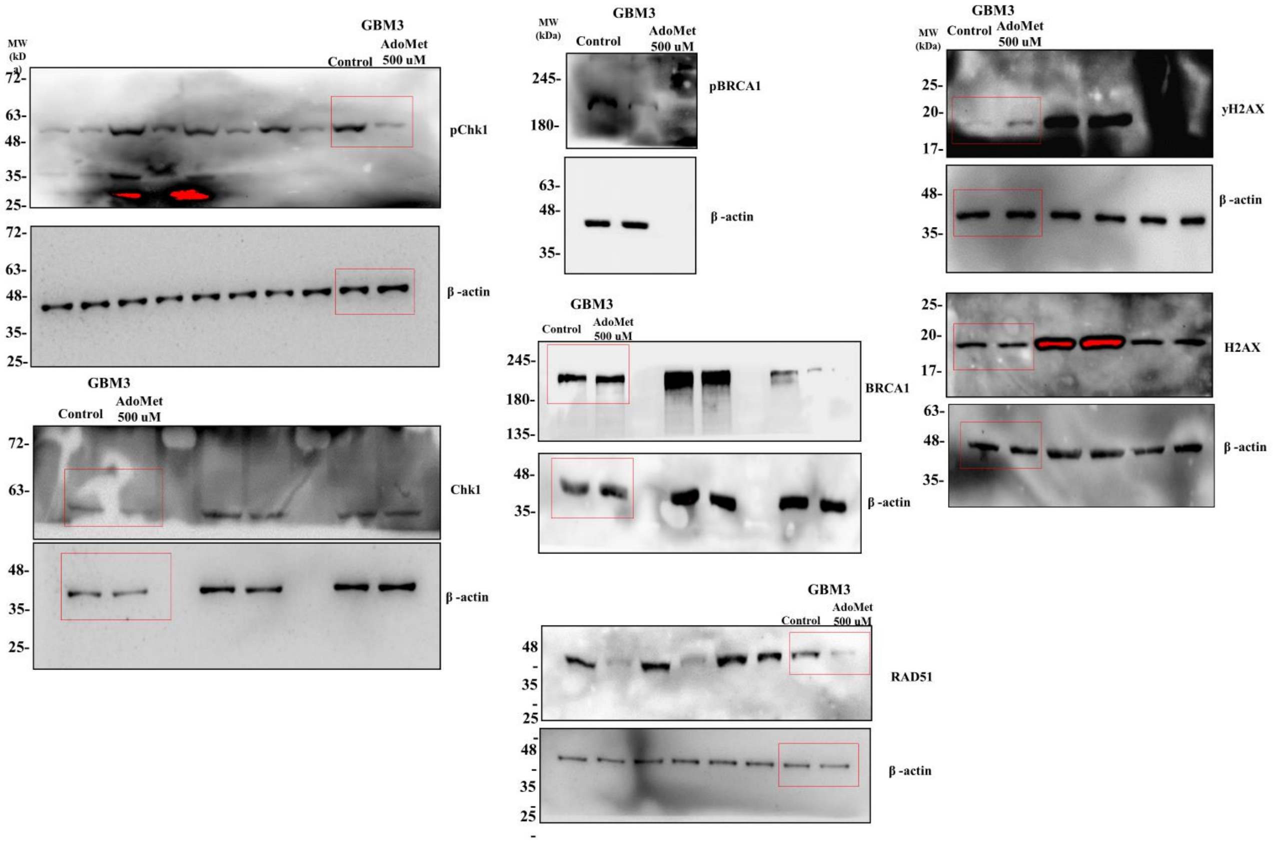

Figure S11: Effect of AdoMet on DNA repair in GBM3 primary cell line.

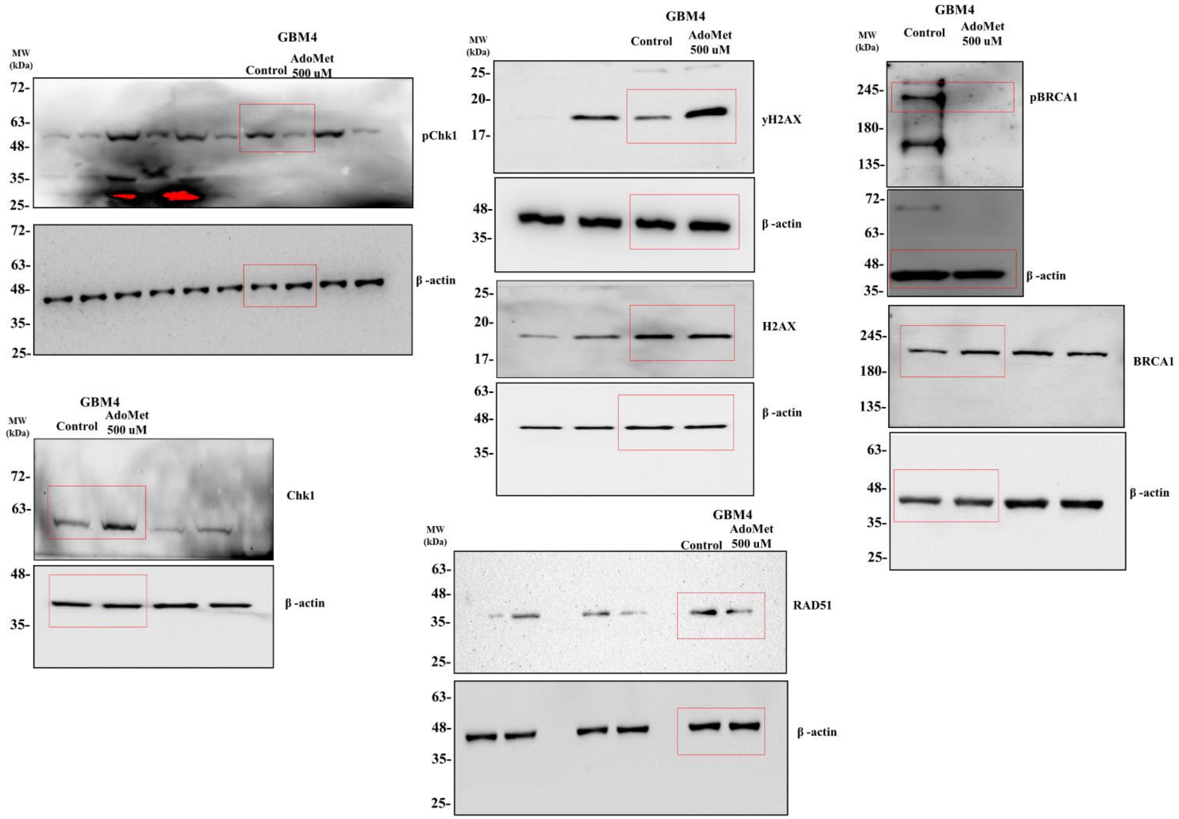

Figure S12: Effect of AdoMet on DNA repair in GBM4 primary cell line.
